# Activation of stimulator of interferon genes (STING) and inhibition of vascular endothelial growth factor receptor (VEGFR) by telatinib induce antitumor activity

**DOI:** 10.1016/j.jbc.2025.111038

**Published:** 2025-12-09

**Authors:** Yi Wang, Yanfei Hou, Jing Han, Zhengyin Zhang, Yiyang Cheng, Jiaming Yang, Luqiu Mou, Shilong Fan, Peiyuan Liu, Kehong Chen, Yuanwei Dai, Conggang Zhang

**Affiliations:** 1 School of Pharmaceutical Sciences, State Key Laboratory of Membrane Biology, Tsinghua-Peking Center for Life Sciences, Beijing Frontier Research Center for Biological Structure, Tsinghua University, Beijing, China; 2 SXMU-Tsinghua Collaborative Innovation Center for Frontier Medicine, Shanxi Medical University, Taiyuan, Shanxi, China; 3 Chongqing Key Laboratory of Natural Product Synthesis and Drug Research, School of Pharmaceutical Sciences, Chongqing University, Chongqing, China; 4 Center for Structural Biology, Tsinghua University, Beijing, China; 5 School of Life Sciences, Tianjin University, Tianjin, China; 6 Department of Nephrology, Daping Hospital, Army Medical University, Chongqing Key Laboratory of Precision Diagnosis and Treatment for Kidney Diseases, Chongqing, China

**Keywords:** STING, immunotherapy, telatinib

## Abstract

The cGAS-STING signaling pathway is a crucial innate immune pathway that senses cytosolic DNA. Pharmacological activation of the cGAS-STING pathway might be a promising strategy for cancer immunotherapy. Here, we report that the cGAS-STING pathway is a new target of telatinib, an orally available vascular endothelial growth factor receptor 2 (VEGFR2) inhibitor that has been investigated in clinical trials. In this study, we demonstrated that telatinib induced innate immune responses in a STING-dependent manner. In addition, we determined the crystal structure of STING bound to a telatinib analog, revealing the molecular interactions underlying STING activation. Moreover, we showed that telatinib-mediated STING activation contributed to the antitumor effects in tumor-bearing mouse models. In summary, our results reveal that telatinib, a previously identified VEGFR2 inhibitor, activates STING signaling, highlighting its potential in cancer immunotherapy.

The recognition of aberrant cytosolic DNA by the cyclic GMP-AMP synthase (cGAS)- stimulator of interferon genes (STING) signaling pathway is a critical part of the innate immunity (1). STING, an endoplasmic reticulum (ER)-localized transmembrane adaptor protein, plays an indispensable role in responding to aberrant cytosolic DNA (2, 3, 4, 5). Upon recognition of cytosolic double-stranded DNA (dsDNA) in the cytosol, the DNA sensor cGAS catalyzes the synthesis of cyclic GMP-AMP (cGAMP), which is the second messenger of the cGAS-STING signaling and binds with the adaptor protein STING (5, 6, 7, 8, 9, 10, 11). After its activation by cGAMP, STING undergoes conformational changes and trafficking from the ER to the Golgi, where STING acts as a platform to recruit TANK-binding kinase 1 (TBK1) and is phosphorylated by TBK1 (10, 12, 13, 14). Following STING phosphorylation, transcription factor IFN regulatory factor 3 (IRF3) is recruited by the STING-TBK1 complex and phosphorylated by TBK1 (13). Phosphorylated IRF3 then translocates into the nucleus and induces the expression of type I interferons (IFNs), such as IFN-β, which further triggers the transcription of interferon-stimulated genes (ISGs) (2, 15). In addition, STING activation also induces NF-κB-dependent proinflammatory cytokine expression (15, 16).

STING-mediated downstream type I IFN expression and proinflammatory cytokine production have potential antitumor effects, such as promoting antigen presentation by dendritic cells (DCs) and the priming of CD8^+^ T cells (17, 18, 19, 20, 21). Therefore, activation of the cGAS-STING pathway has emerged as a promising strategy for cancer immunotherapy. In recent years, numerous STING agonists have been reported (22, 23, 24, 25, 26, 27, 28, 29, 30, 31, 32, 33, 34, 35). The first class of STING agonists is cyclic dinucleotides (CDNs) and their analogs (*e.g.*, MIW815 and MK-1454) (26, 27, 31, 32, 33). Moderate clinical responses have been observed in patients treated with cGAMP analogs (36). Meanwhile, more and more non-CDN STING agonists have been discovered and demonstrated to exhibit antitumor activity in preclinical tumor models (22, 23, 24, 25, 28, 29, 30). Nevertheless, the clinical development of STING agonists, both CDN and non-CDN compounds, remains challenging and needs to achieve more satisfactory outcomes. In addition, many STING agonists are administered by intratumoral injection, which is a clinically unfeasible administration route. Moreover, safety is always the highest priority in pharmaceutical development. The toxic effects (*e.g.*, hepatic toxicity and T cell death) induced by STING agonists need to be considered in the development (37, 38, 39, 40). Therefore, it is necessary to develop STING agonists with high efficacy and good safety profiles.

In this study, we reported the discovery of a non-CDN STING agonist, telatinib, which was previously developed as a VEGFR2 inhibitor. We demonstrated that telatinib activated the cGAS-STING signaling and induced potent STING-dependent innate immune responses. Mechanistic studies using Surface Plasmon Resonance (SPR) assay confirmed the direct binding between telatinib and STING, and the co-crystal structure showed that the analog of telatinib bound to the ligand-binding pocket of STING. Furthermore, the investigations in tumor-bearing mouse models showed that telatinib inhibited tumor growth and prolonged survival, which was impaired in *Sting1*^*−/−*^ mice. Collectively, our findings indicated that telatinib could activate STING signaling, which was important for its antitumor effect.

## Results

### Telatinib induces innate immune responses

**Figure 1:**
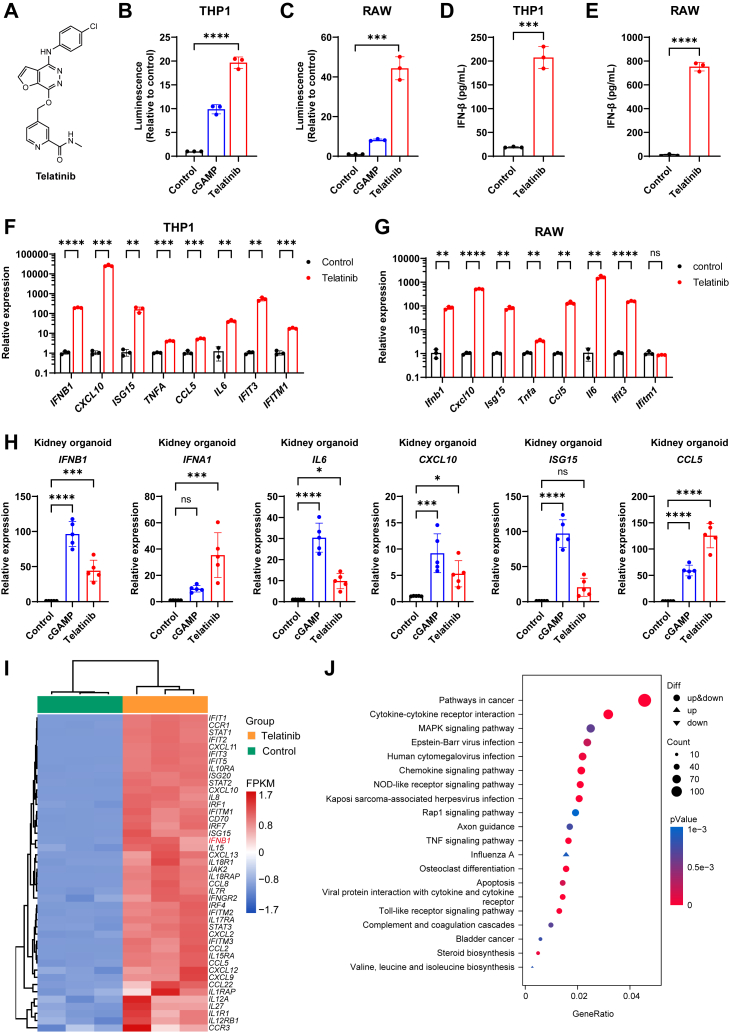
**Telatinib activates the type I interferon response.***A*, structure of telatinib. *B*, luciferase activity of THP1-Lucia ISG cells treated with cGAMP (1 μM) or telatinib (20 μM) for 24 h. The luminescence data are normalized to the control group. *C*, luciferase activity of RAW-Lucia ISG cells treated with cGAMP (2 μM) or telatinib (30 μM) for 24 h. The luminescence data are normalized to the control group. *D* and *E*, IFN-β production in THP1-Lucia ISG cells (D) and RAW-Lucia ISG cells (*E*) treated with telatinib (25 μM) for 12 h. *F*, q-PCR analysis of target gene expression in THP1-Lucia ISG cells treated with telatinib (25 μM) for 12 h (n = 3 biological replicates). *G*, q-PCR analysis of target gene expression in RAW-Lucia ISG cells treated with telatinib (25 μM) for 6 h (n = 3 biological replicates). *H*, q-PCR analysis of target gene expression in kidney organoids stimulated with cGAMP (4 μM) or telatinib (25 μM) (n = 5 biological replicates). *I* and *J*, RNA-seq analysis of THP1-Lucia ISG cells treated with or without telatinib (25 μM) for 12 h. *I*, Heatmap showing the expression of interferon-stimulated genes. *J*, KEGG enrichment of DEGs. ∗*p* < 0.05, ∗∗*p* < 0.01; ∗∗∗*p* < 0.001; ∗∗∗∗*p* < 0.0001; NS, not significant (*p* > 0.05). Data are shown as the mean ± SD. Statistical significance was determined using an unpaired *t* test.

To identify activators of the innate immune response, we performed high-throughput screening using THP1-Lucia ISG cells (Fig. S1*A*), a human monocyte cell line containing an interferon regulatory factor (IRF)-inducible luciferase (Lucia) reporter (23, 24). Using this cell-based functional assay, we found that telatinib induced a robust increase in luciferase activity (Fig. 1, *A* and *B*). Consistently, telatinib also stimulated reporter activity in RAW-Lucia ISG cells (Fig. 1*C*), a murine macrophage cell line with IRF-inducible Lucia luciferase reporter, suggesting that telatinib could activate the IRF immune response in both human and mouse immune cell lines. This immune activation induced by telatinib was further validated by detecting the expressions of IFN-β and proinflammatory factors. Indeed, telatinib treatment significantly upregulated the protein levels of IFN-β (Figs. 1, *D* and *E*, and S1*B*) and the mRNA levels of IFN-β, CXCL10, ISG15, IL6, and IFIT3 (Figs. 1, *F* and *G*, and S1*C*) in THP1 cells, RAW cells, and human peripheral blood mononuclear cells (hPBMCs). Besides the effects in the cell lines, telatinib also increased the expression of IFN-β and proinflammatory factors in human kidney organoids (Fig. 1*H*).

To further investigate the effects of telatinib, we performed RNA sequencing (RNA-seq) analysis on THP1 cells stimulated with telatinib. RNA-seq analysis revealed 2238 differentially expressed genes (DEGs) in telatinib-treated cells compared to untreated THP1 cells (Fig. S1*D*). Among the upregulated DEGs, a substantial number were involved in IFN-β signaling (Fig. 1*I*). KEGG pathway enrichment analysis of the DEGs showed that telatinib strongly affected innate immune signaling pathways, especially pathways in cancer and cytokine-cytokine receptor activation (Fig. 1*J*). Furthermore, gene set enrichment analysis (GSEA) also identified that some STING-associated pathways, including the IFN-α response and IL-6-JAK-STAT3 signaling pathways, were upregulated by telatinib treatment (Fig. S1*E*). Together, these results demonstrate that telatinib robustly induces the type I interferon immune response.

### Telatinib activates innate immunity in a STING-dependent manner

**Figure 2:**
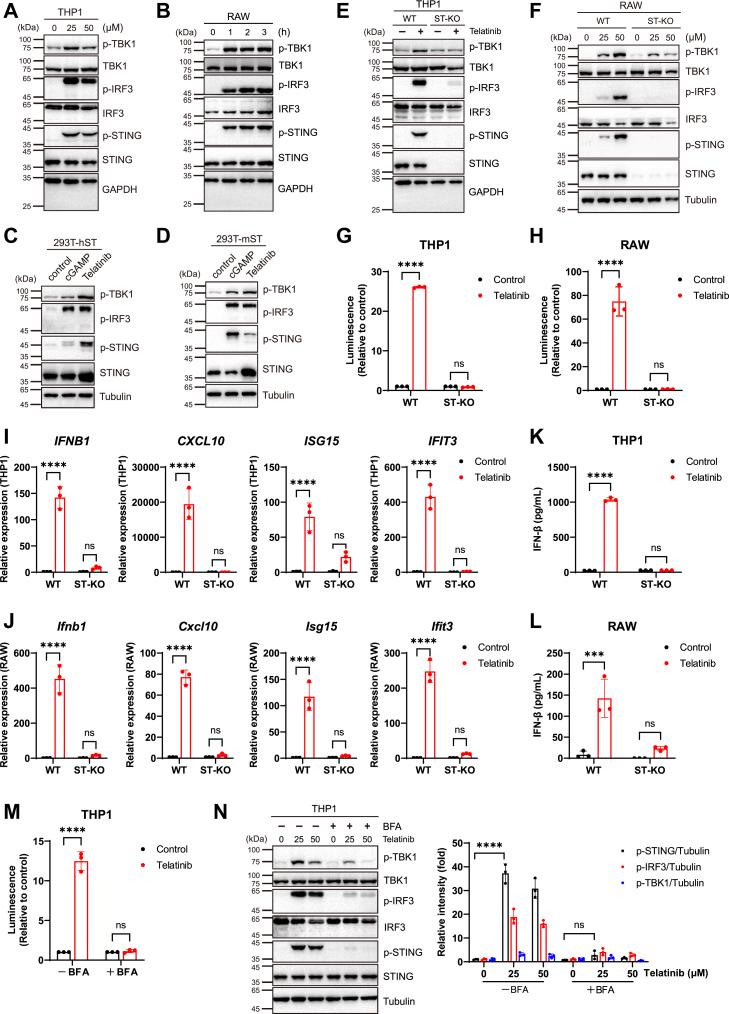
**Telatinib activates the cGAS-STING pathway in a STING-dependent manner.***A*, Western blot analysis of THP1-Lucia ISG cells treated with telatinib (0, 25, 50 μM) for 2 h. (*B*) Western blot analysis of RAW-Lucia ISG cells treated with telatinib (25 μM) for the indicated time. *C* and *D*, Western blot analysis of HEK293 T cells stably expressing human STING (hSTING) (*C*) or mouse STING (mSTING) (*D*) treated with cGAMP (1 μM) or telatinib (25 μM). *E*, Western blot analysis of WT and ST-KO THP1-Lucia ISG cells treated with or without telatinib (25 μM) for 2 h. *F*, Western blot analysis of WT and ST-KO RAW-Lucia ISG cells treated with telatinib (0, 25, 50 μM) for 2 h. *G* and *H*, Luciferase activity of WT and ST-KO THP1-Lucia ISG cells (*G*) and RAW-Lucia ISG cells (*H*) treated with telatinib (20 μM) for 24 h. The luminescence data are normalized to the control group. *I*, q-PCR analysis of target gene expression in WT and ST-KO THP1-Lucia ISG cells treated with telatinib (25 μM) for 12 h (n = 3). *J*, q-PCR analysis of target gene expression in WT and ST-KO RAW-Lucia ISG cells treated with telatinib (50 μM) for 12 h (n = 3 biological replicates). *K*, IFN-β production in WT and ST-KO THP1-Lucia ISG cells treated with telatinib (25 μM) for 12 h. *L*, IFN-β production in WT and ST-KO RAW-Lucia ISG cells incubated with telatinib (50 μM) for 12 h. *M*, luciferase activity of THP1-Lucia ISG cells treated with telatinib (30 μM) in the presence or absence of BFA (1 μM) for 24 h. The luminescence data are normalized to the control group. *N*, *left*: Western blot analysis of THP1-Lucia ISG cells treated as indicated for 2 h. *Right*: Western blotting quantification was performed using ImageJ (n = 3 biological replicates). All data are representative of three independent experiments. ∗∗∗*p* < 0.001; ∗∗∗∗*p* < 0.0001; NS, not significant (*p* > 0.05). Data are shown as the mean ± SD. Statistical significance was determined using two-way ANOVA.

We then investigated whether the cGAS-STING pathway is involved in telatinib-stimulated immune response. Western blotting analysis showed that telatinib induced the phosphorylation of STING, TBK1, and IRF3 proteins in THP1 cells, RAW cells, and PBMCs (Figs. 2, *A* and *B* and S2, *A*–*C*), indicating that telatinib activated the STING-TBK1-IRF3 signaling cascades in both human and mouse cells. To exclude the possibility that STING activation is induced by the release of nuclear or mitochondrial DNA, human or mouse STING (hSTING or mSTING) was stably expressed in the HEK293 T cell line, which lacks endogenous STING expression. In the absence of cGAS, phosphorylation of STING, TBK1, and IRF3 was still observed after telatinib treatment (Figs. 2, *C* and *D* and S2, *D* and *E*). In addition, telatinib retained its ability to activate the STING pathway in cGAS-knockout (cGAS-KO) THP1 cells, confirming its cGAS-independent activity (Fig. S2*J*).

To further examine the role of the STING pathway in telatinib-mediated immune response, we generated STING-knockout (ST-KO) THP1 cells and RAW cells (Fig. S2, *F* and *G*). As expected, STING deficiency abolished the activation of the cGAS-STING pathway in THP1 cells and RAW cells with cGAMP or telatinib treatments (Figs. 2, *E*–*H* and S2, *F*–*I*). In addition, telatinib failed to induce the expression of IFN-β and proinflammatory cytokines in STING-knockout THP1 cells and RAW cells (Fig. 2, *I*-*L*), demonstrating that STING is critical in immune responses induced by telatinib. Consistent with these results, telatinib was unable to induce phosphorylation of TBK1 and IRF3 in HEK293 T cells lacking STING expression (Fig. S2, *K* and *L*). Moreover, wild-type (WT) HEK293 T cells were transfected with IFN-β luciferase reporter plasmids with or without STING constructs. The results showed that telatinib induced IFN-β expression in the presence of STING, and no immune response was detected in STING-deficient cells (Fig. S2, *M* and *N*). Previous studies have shown that STING trafficking from the ER to the Golgi is required for STING pathway activation. Treatment with brefeldin A (BFA), an inhibitor of ER-to-Golgi trafficking, suppressed the STING activation induced by telatinib (Fig. 2, *M* and *N*), further indicating that STING plays a primary role in the induction of immune signaling by telatinib.

Collectively, these results demonstrate that telatinib induces immune responses in a STING-dependent pattern.

### Crystal structure of the STING-telatinib analog complex reveals a potential mechanism of STING activation by telatinib

**Figure 3:**
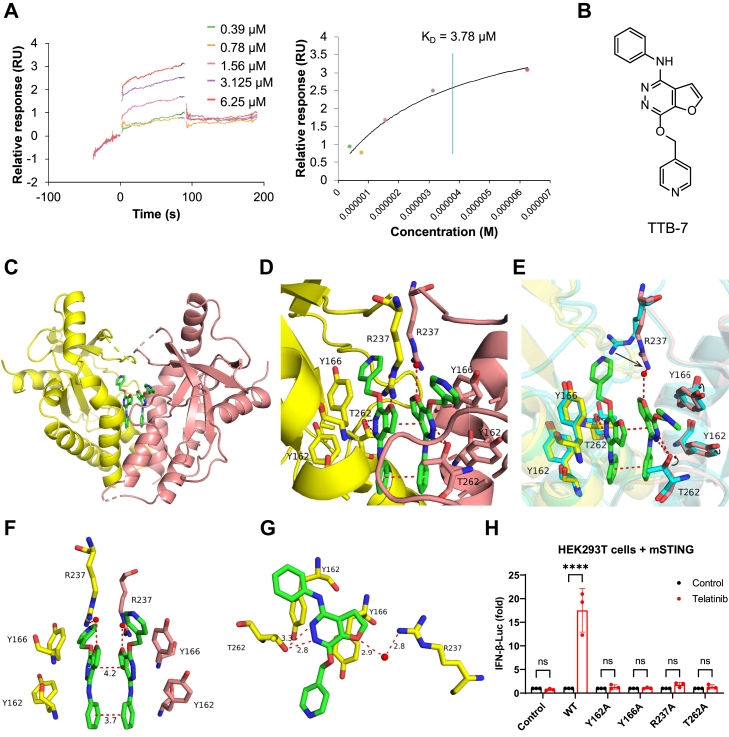
**STING interacts with telatinib and its analog TTB-7.***A*, the binding of telatinib to mSTING (154–340) was tested by SPR assay. *B*, structure of TTB-7. *C*, structure of TTB-7 bound to mSTING (residues 154–340). The mSTING dimer is shown in ribbon representation, and TTB-7 is shown in stick representation. *D*–*G*, details of the intermolecular contacts in the binding sites of TTB-7-bound mSTING complex. Residues from individual monomers of the TTB-7-bound mSTING dimer are shown in *yellow* and *red*. Residues from the apo mSTING structure (PDB: 4KC0) are shown in *blue*. The *arrows* in (*E*) depict the side-chain movements of key residues. *H*, HEK293 T cells were transfected with WT or mutated mSTING plasmids, then incubated with or without telatinib (25 μM) for 24 h, followed by measurement of luciferase activity. The luminescence data are normalized to the control group. ∗∗∗∗*p* < 0.0001; NS, not significant (*p* > 0.05). Data are shown as the mean ± SD. Statistical significance was determined using two-way ANOVA.

To elucidate the mechanism of telatinib-induced STING activation, we performed binding assays using the STING ligand-binding domain (residues 154–340). Surface Plasmon Resonance (SPR) assay demonstrated direct binding between telatinib and mSTING with a K_D_ of 3.78 μM (Fig. 3*A*). We then tried to characterize the STING-telatinib complex through the crystallization approach. However, the STING-telatinib complex failed to crystallize. Subsequently, we synthesized a series of telatinib analogs for crystallization screening (Fig. S3, *A* and *B*). Finally, we determined the crystal structure of STING in complex with one analog, TTB-7, at a resolution of 1.92 Å, although TTB-7 was found to be unable to activate STING signaling (Figs. 3, *B* and *C*, and S3*C*). The structure showed that the STING dimer is closed and compact, with two TTB-7 molecules buried in the ligand-binding domain (LBD) of the STING dimer (Fig. 3, *C*–*E*). At the dimer interface, the two TTB-7 molecules interact *via* π−π stacking between their aromatic rings and are bracketed by the side chains of Y162 and Y166 (Fig. 3*F*). Each TTB-7 molecule forms hydrogen bonds with water molecules and the side chains of surrounding residues. The nitrogen of furo[2,3-days]pyridazine interacts with the hydroxyls of T262 (Fig. 3*G*). The side chains of R237 protrude into the binding pocket and interact with the oxygen of furo[2,3-days]pyridazine through water-mediated hydrogen bonds (Fig. 3*G*). To validate whether telatinib has the same binding pattern as TTB-7, we mutated key residues in mSTING (Y162A, Y166A, R237A, and T262A) and transfected HEK293T cells with WT or mutant STING constructs to perform IFN-β luciferase reporter assays. The mutagenesis results showed that mutations of these residues abolished the STING activation stimulated by telatinib (Figs. 3*H* and S3*D*), suggesting the importance of these key residues in the telatinib-induced STING signaling.

We further compared and analyzed the TTB-7–bound STING structure with mouse STING in complex with c-di-GMP (26, 31), cGAMP (10), DMXAA (30, 41), and SR-717 (23). The TTB-7–bound STING structure closely resembled the apo mSTING (4KC0) and the c-di-GMP-bound mSTING (4KBY) (26) (Fig. S4, *A*, B, and F). STING dimer adopts a closed conformation with two loops forming the ‘lid’ region. The lid residues consist of residues M223 to Q227 and residues R237 to N241. This conformation is very compact, with a distance of approximately 30 Å between the tips of α2 helices. By contrast, other ligand-bound mSTING structures exhibited distinct conformations, with α2-helix tip distances of 38 Å in the structure of cGAMP-bound mSTING (4LOJ), 42 Å in the structure of DMXAA-bound mSTING (4LOL), and 37 Å in the structure of SR-717-bound mSTING (6XNN) (Fig. S4, *C*–*E*).

**Figure 4:**
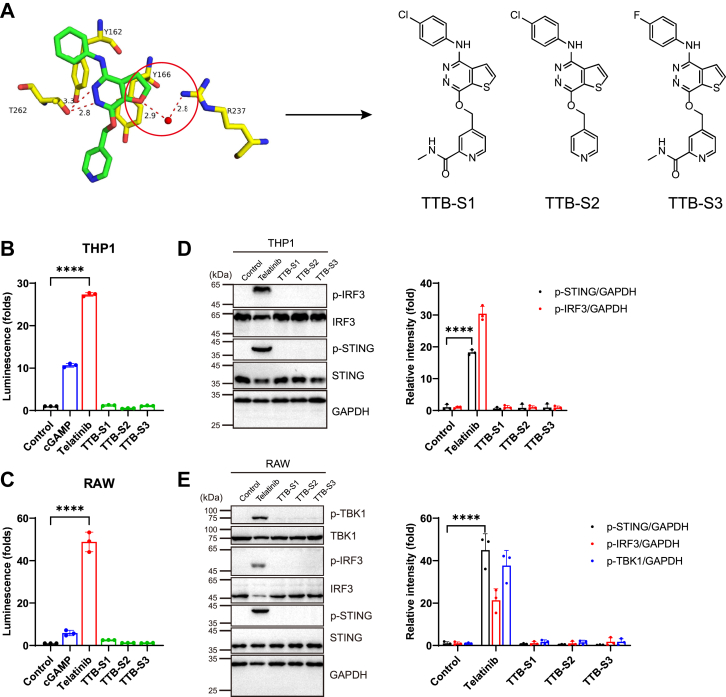
**Structure and activity validation of telatinib analogs.***A*, Chemical structures of three telatinib analogs. *B* and *C*, luciferase activity of THP1-Lucia ISG (*B*) and RAW-Lucia ISG cells (*C*) treated with cGAMP (1 μM), telatinib (20 μM), TTB-S1 (20 μM), TTB-S2 (20 μM), and TTB-S3 (20 μM) for 24 h. The luminescence data are normalized to the control group. *D* and *E*, *Left*: Western blot analysis of THP1-Lucia ISG (*D*) and RAW-Lucia ISG cells (*E*) treated with telatinib (25 μM), TTB-S1 (25 μM), TTB-S2 (25 μM), and TTB-S3 (25 μM) for 2 h. *Right*: Western blotting quantification was performed using ImageJ (n = 3 biological replicates). All data are representative of three independent experiments. ∗∗∗∗*p* < 0.0001. Data are shown as the mean ± SD. Statistical significance was determined using an unpaired *t* test.

Based on the structure of the STING–TTB-7 complex, we designed telatinib analogs to investigate the functional role of specific intermolecular interactions. The interaction between R237 and the furo[2,3-days]pyridazine of TTB-7 suggests that the oxygen in the furo[2,3-days]pyridazine might be critical for telatinib-mediated STING activation. To test this hypothesis, we substituted the oxygen atom with sulfur and generated three sulfur-substituted analogs—TTB-S1, TTB-S2, and TTB-S3 (Fig. 4*A*). As expected, none of these three compounds activated STING signaling (Fig. 4, *B*–*E*). To exclude the possibility that the loss of activity resulted from impaired cellular permeability, THP1 and RAW cells were treated with telatinib or TTB-S1-S3 in the presence or absence of the membrane-permeabilizing protein perfringolysin O (PFO). However, TTB-S1-S3 still failed to induce STING activation even with PFO treatment (Fig. S3*E*), suggesting that the inactivity was not due to impaired cell permeability. Therefore, we propose that the sulfur atom crowds out the water molecule due to its larger atomic size and abolishes the hydrogen bonds with R237, indicating that the interaction between R237 and the oxygen of furo[2,3-days]pyridazine bridged by a water molecule is critical for the function of telatinib.

Taken together, we solved the crystal structure of the STING–TTB-7 complex. Further research is required to optimize telatinib based on this structural information to develop more potent telatinib analogs that activate STING signaling.

### The antitumor activity of telatinib is dependent on STING

**Figure 5:**
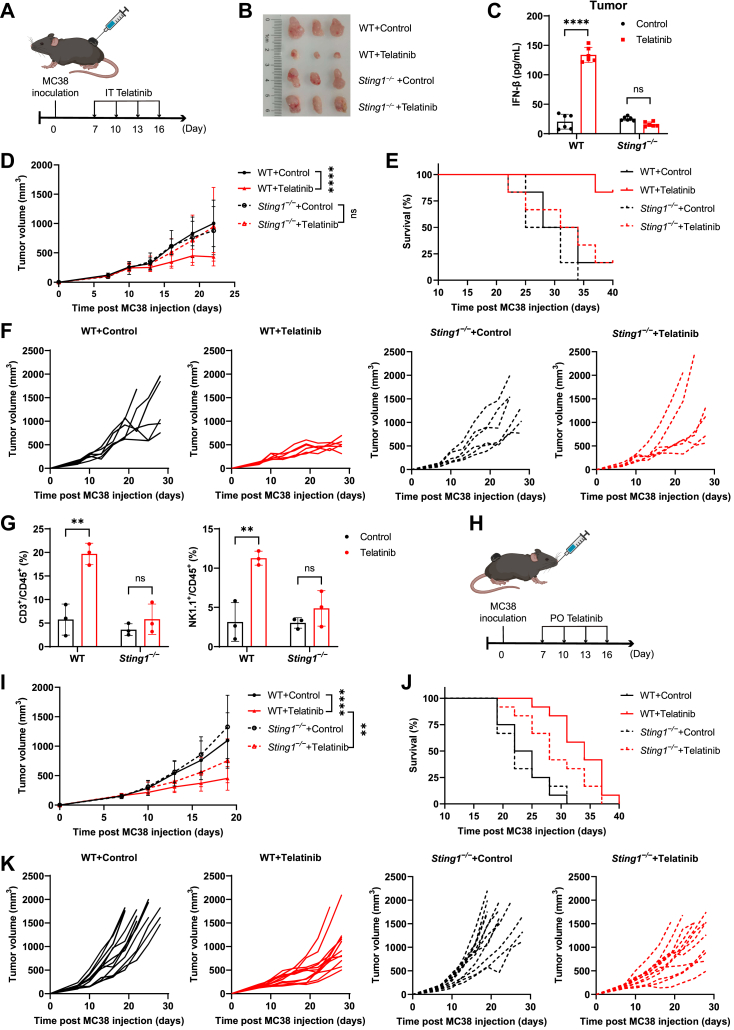
**Telatinib shows STING-dependent antitumor activity.***A*, schematic of dosing strategy by intratumoral (IT) administration of telatinib (600 μg) in WT or *Sting1*^*−/−*^ MC38 tumor-bearing mice (n = 6). *B*, representative image of isolated tumors from MC38 tumor-bearing mice treated as described in (*A*). *C*, IFN-β levels in B16 tumors (n = 6) from WT and *Sting1*^*−/−*^ mice 8 h after IT injection of telatinib (600 μg). *D*–*F*, tumor growth curve (*D*), survival curve (*E*), and individual tumor growth (*F*) of MC38 tumor-bearing mice treated as described in (*A*). *G*, quantification of T cells and NK cells in tumors of WT or *Sting1*^*−/−*^ MC38 tumor-bearing mice (n = 3) (administered as described in *A*) by flow cytometry. *H*, schematic of dosing strategy by oral administration (PO) of telatinib (600 μg) in WT or *Sting1*^*−/−*^ MC38 tumor-bearing mice (n = 12). *I*–*K*, tumor growth curve (*I*), survival curve (*J*), and individual tumor growth (*K*) of MC38 tumor-bearing mice treated as described in (*H*). ∗∗*p* < 0.01; ∗∗∗∗*p* < 0.0001; NS, not significant (*p* > 0.05). Data are shown as the mean ± SD. Statistical significance was determined using two-way ANOVA.

Previous studies and our results collectively demonstrate that telatinib is an orally available vascular endothelial growth factor receptor 2 (VEGFR2) inhibitor with antitumor activity (Figs. S5, *A*–*C*, and S6, *E* and *F*) (42, 43, 44). To evaluate whether telatinib-mediated STING activation contributes to its antitumor activity, we established B16 and MC38 syngeneic tumor models in WT and *Sting1*^*−/−*^ mice (Figs. 5, *A* and *H*, and S5*D*). Telatinib was administered *via* intratumoral (IT) or oral (PO) routes and exhibited favorable pharmacokinetic properties (Fig. S6, *G* and *H*). The results showed that telatinib suppressed tumor growth and prolonged survival in WT tumor-bearing mice, but the antitumor effects of telatinib were impaired in *Sting1*^*−/−*^ tumor-bearing mice (Figs. 5, *B*, *D*–*F*, *I*–*K*, and S5, *D*–*F*), providing evidence that the STING pathway plays an important role in the antitumor activity of telatinib. Interestingly, the therapeutic effects of telatinib on *Sting1*^*−/−*^ mice were influenced by administration routes. In *Sting1*^*−/−*^ mice intratumorally administered with telatinib, STING deficiency completely abolished its antitumor activity (Fig. 5, *D*–*F* and S5D-F); while for *Sting1*^*−/−*^ mice treated with telatinib by oral administration, telatinib still showed antitumor activity, although its antitumor effect was attenuated compared to WT mice (Fig. 5, *I*–*K*). The antitumor effects in *Sting1*^*−/−*^ mice orally treated with telatinib are most likely attributable to the angiogenesis inhibition induced by telatinib. In addition, intratumoral injection of telatinib increased the level of IFN-β in the B16 tumors in a STING-dependent manner (Fig. 5C). We also investigated the impact of telatinib on tumor-infiltrating lymphocytes in MC38 tumor tissues from mice administered intratumorally by flow cytometry. Telatinib increased the proportions of natural killer (NK) cells and T cells among tumor-infiltrating lymphocytes in a STING-dependent manner (Fig. 5*G*), supporting that immune responses contributed to the antitumor effects of telatinib *in vivo*. In addition, mice treated with telatinib showed no body weight loss, indicating a good safety profile (Fig. S6, *A*–*D*). These results are consistent with previous pharmacodynamic and pharmacokinetic studies that telatinib is safe and well-tolerated up to 1500 mg twice daily in a phase I dose-escalation study (42). In summary, these results demonstrate that telatinib has potential in antitumor immunotherapies through activating STING signaling.

## Discussion

In this study, we identified telatinib as a novel non-CDN STING agonist through high-throughput screening. Our results demonstrated that telatinib activated the STING pathway in both human and murine cells, inducing downstream immune responses in a STING-dependent manner. In accordance with this, RNA-seq analysis revealed that telatinib treatment upregulated IFN-β signaling-related genes and pathways. Mechanistically, the crystal structure of STING with TTB-7 and the SPR assay provide evidence that STING is the target of telatinib and provide instructions for further structure optimization of the lead compound. Notably, TTB-7-bound mSTING adopts a rare compact conformation with only 30 Å between the tips of α2 helices, which is distinct from the more open conformation observed in cGAMP-bound mSTING. One possible explanation for the different distance between the tips of α2 helices is that the residues M223 to N241 form a four-strand β-sheet lid over the binding pocket in cGAMP-bound mSTING. In comparison, residues N228 to N236 were disordered in TTB-7-bound mSTING, enabling TTB-7-bound mSTING to form a more compact conformation. Although the significance of the “open” or “closed” conformation is not sufficiently explored, existing evidence suggests that the conformational change contributes to differences in the potency of the STING pathway and the formation of STING oligomers (45, 46, 47). Using cryo-EM to investigate STING oligomeric assemblies (with different agonists) with different closed and open conformation of LBD will help to characterize the precise mechanisms.

While telatinib has previously been characterized as an angiogenesis inhibitor in clinical trials (42, 43, 44), our data uncovered a new function of telatinib in activating the STING pathway, which also contributes to its antitumor efficacy. This conclusion is supported by the results that the antitumor effects of telatinib were impaired in *Sting1*^*−/−*^ mice, although orally administered telatinib retained attenuated antitumor effects in *Sting1*^*−/−*^ mice, which might be attributed to its VEGFR2 inhibitor function. Furthermore, the increased proportion of T cells and NK cells among tumor-infiltrating lymphocytes supported that telatinib administered intratumorally induced antitumor immune activity. In addition, it has previously been reported that STING activation inhibits tumor angiogenesis properties, which could synergize with telatinib in the inhibition of VEGFR2 (48). Therefore, telatinib exerts antitumor effects by simultaneously inhibiting VEGFR2 and activating the STING pathway. However, oral delivery carries a potential risk of eliciting systemic inflammation and excessive cytokine release (39, 49). In contrast, intratumoral injection can minimize systemic adverse effects and induce more robust local STING activation, though its clinical application is limited by tumor accessibility. Therefore, the development of advanced drug delivery systems (*e.g.*, nanoparticles) is crucial to improve solubility and enhance tumor-specific targeting for better safety and efficacy.

Since STING agonists have potential in antimicrobial and antitumor treatment, many STING agonists have been discovered with diverse targeting mechanisms. Some of these agonists could only activate human or mouse STING. For example, DMXAA and CMA are specific mouse STING agonists (41, 50, 51), whereas G10 and NVS-STG2 are human STING-specific agonists (28, 52, 53). In this study, we showed that telatinib activated both human and mouse STING. To understand the mechanism of STING activation by agonists, multiple STING agonists have been structurally characterized to reveal their binding modes with STING. CDNs and their analogs bind STING LBD and activate STING (9, 41, 54, 55). Moreover, non-CDN STING agonists, including amidobenzimidazole (ABZI)-based compounds, SR-717, and MSA-2, also bind STING LBD and exhibit antitumor activity (22, 23, 24). By contrast, two other compounds, C53 and NVS-STG2, bind to the transmembrane domain of STING and induce STING oligomerization (25, 28). Together, the efforts to develop STING agonists and determine their molecular mechanisms could not only increase the choices during STING activation but also help to deepen our understanding of STING signaling. Here, we report telatinib as a new activator of STING. Notably, one significant advantage of this new STING activator is its safety, which has been evaluated in multiple animal studies and clinical trials (42, 43). As the safety issue is one of the most important concerns in the clinical trials for STING agonists, including STING agonist antibody-drug conjugates (ADC) and other combination strategies, the discovery of telatinib would be a helpful complement to the field of STING agonists. Moreover, recent studies report that STING activation can alleviate pain in the nervous system (56, 57, 58). It would be interesting to see whether the administration of telatinib and other STING agonists would have therapeutic benefits for pain management.

Finally, while STING agonists show potential in antitumor immune responses, many questions remain to be answered in STING-mediated cancer treatment. A key issue is elucidating the mechanisms of tumor immunosuppression, which may be a critical reason for the failure of clinical trials in STING agonist-mediated cancer immunotherapies. For example, IL-6 exerts a double-edged sword function in cancer therapy. IL-6 promotes T cell survival and infiltration in tumors (59, 60), while also promoting tumor cell proliferation, survival, and metastasis (61). Recent studies have shown that IL-6 expression (40), type I IFN-independent NF-кB activity (62), and IL-35 expression by regulatory B cells (63) may contribute to tumor resistance to STING monotherapy (64). Conversely, several studies report that immune activation induced by STING agonists exerts antitumor effects (18, 35, 36, 65, 66, 67, 68, 69). Studies show that IL-18 secretion induced by STING agonists enhances the therapeutic efficacy of CAR-T(18); IL-2 and STING agonists regulate the activation of NK cells and inhibit tumorigenesis (69); a combination of STING agonists with other existing cancer therapies also has potential in antitumor treatment (70, 71). Thus, a more comprehensive understanding of telatinib, as well as its combination with other chemotherapy or immunotherapy, such as anti-PD-1 treatment or IL-6R inhibitors, may contribute to a promising and safe antitumor therapeutic strategy.

## Experimental procedures

### Antibodies and reagents

The antibodies used in this study were as follows: rabbit anti-STING (D2P2F) (Cat# 13647; RRID: AB_2732796), anti-phospho-IRF-3 (Ser396) (4D4G) (Cat# 4947; Lot: 13; RRID: AB_823547), anti-phospho-STING (Ser366) (D7C3S) (Cat# 19781; Lot: 3; RRID: AB_2737062), anti-phospho-TBK1/NAK (Ser172) (D52C2) (Cat# 5483; Lot: 13; RRID: AB_10693472), anti-phospho-STING Ser366 (D8F4W) antibody (Cat# 72971; Lot: 3; RRID: AB_2799831), and Rabbit monoclonal anti-GAPDH (14C10) antibody (Cat# 2118; RRID: AB_561053) were purchased from Cell Signaling Technology; mouse anti-β-Tubulin antibody (Cat# M20005; RRID: AB_2920648) was purchased from Abmart; anti-TBK1 antibody (Cat# ab40676; Lot: 1094437-4; RRID: AB_776632) was purchased from Abcam. Rabbit polyclonal anti-IRF3 antibody (Cat# 11312-1-AP; RRID: AB_2127004) was purchased from Proteintech. Telatinib (#HY-10527), DMXAA (#HY-10964), and Brefeldin A (BFA) (#HY-16592) were purchased from MedChemExpress. VEGFR2 (KDR) Kinase Assay Kit (40325) was purchased from BPS Bioscience. Collagenase IV (C5138) was purchased from Sigma. DNase I (GMP-E127) was purchased from Novoprotein.

### Mouse studies

C57BL/6 WT mice and *Sting1*^*−/−*^ mice were obtained from Tsinghua University. All mice were immunocompetent and maintained on a 12/12-h light/dark cycle, 22 to 26 °C, 30 to 70% relative humidity with sterile pellet food and water ad libitum. 6-8-week-old male and female mice were subcutaneously implanted with MC38 (7 × 10^5^) cells or B16 (2 × 10^5^) cells and randomly assigned to the control group and telatinib group. On days 7, 10, 13, and 16 after tumor cell implantation, mice are administered orally or intratumorally with telatinib. On day 14, tumors were isolated from mice and used for flow cytometry. The tumor volume and body weight were measured every 3 days from day 7. Tumor volume was calculated using the formula: tumor volume = length x width^2^/2. Mice were euthanized when tumor volume reached 1500 mm^3^. To study the pharmacokinetics of telatinib, WT C57BL/6 mice were dosed with 15 mg/kg telatinib by IT injection or 30 mg/kg telatinib by PO gavage. Plasma and tumor samples were collected at 0, 0.083, 0.25, 0.5, 1, 2, 4, 8, and 12 h. The concentration of telatinib in samples was measured by mass spectrometry. All housing, breeding, and procedures were performed following the regulations in the Guide for the Care and Use of Laboratory Animals issued by the Ministry of Science and Technology of the People’s Republic of China. All animal protocols were approved by the Institutional Animal Care and Use Committees of Tsinghua University.

### Cell culture and stable cell line generation

RAW264.7, THP1, MC38, B16, and HEK293T cells were purchased from ATCC. Human peripheral blood mononuclear cells (hPBMC) were isolated from blood donated by healthy volunteers. All cells were cultured at 37 °C under 5% CO_2_ in a humidified incubator. RAW264.7, MC38, B16, and HEK293T cells were cultured in DMEM (Gibco) supplemented with 10% (v/v) Fetal Bovine Serum (FBS, ExCell), 0.1 mg/ml streptomycin, and 100 U/ml penicillin. THP1 cells were cultured in RPMI-1640 (Gibco) supplemented with 10% (v/v) Fetal Bovine Serum (FBS), 0.1 mg/ml streptomycin, and 100 U/ml penicillin. Human PBMC were cultured in ImmunoCult-XF T Cell Expansion Medium (STEMCELL technologies, 10,981) supplemented with the ImmunoCult Human CD3/CD28/CD2 T Cell Activator (STEMCELL, Catalog #10970).

For stable cell line construction, human STING and mouse STING genes were cloned into pCDH vectors. CRISPR gRNAs targeting human STING (sgRNA: 5′- GGATGTTCAGTGCCTGCGAG-3′) or mouse STING (sgRNA: 5′- ACTCTTCTGCCGGACACTTG-3′) were cloned into LentiCRISPR-V2-puro (RRID: Addgene_52961). The constructs and helper plasmids (psPAX-2 and pMD2.G) were transfected into HEK293T cells using polyethyleneimine (PEI, Polysciences). The medium containing lentiviral particles was collected 48 and 72 h later, and filtered through 0.22 μm filters. RAW264.7, THP1, and HEK293T cells were cultured with the medium in the presence of polybrene overnight, then selected with puromycin (2 μg/ml) or blasticidin (5 μg/ml) for 48 h. The efficiency of gene overexpression or deletion was confirmed by Western blot.

### Human kidney organoids

Cancer tissues from nephrectomies of renal cancer patients were collected at the Daping Hospital. The study was approved by the Ethics Committee of Daping Hospital (No.2020248). Written informed consent was obtained from the patients and/or their authorized representatives. This study complies with the ethical principles of Declaration of Helsinki.

### High-throughput screening

THP1-Lucia ISG cells at a density of 3 × 10^6^ cells/ml were incubated with test compounds (10 μM) of MCE Kinase Inhibitor Library in 384-well plates and cultured at 37 °C under 5% CO_2_ for 24h. 50 μl substrate buffer (50 mM HEPES pH 7.0, 50 mM NaCl, 0.05% CHAPS, 10 mM EDTA, 1 μM Coelenterazine) was added to each well, then luminescence was detected using a Cytation Cell Imaging Reader (BioTek’s).

### Luciferase reporter assay

THP1-Lucia ISG cells and RAW-Lucia ISG cells at a 3 × 10^6^ cells/ml density were incubated with cGAMP or telatinib in 96-well plates. After 24 h incubation, the supernatant was transferred to a 96-well white plate and mixed with substrate buffer. Luminescence was detected using a Cytation Cell Imaging Reader.

HEK293T cells were seeded onto the 96-well plates and transiently co-transfected with the secretory IFNβ-luciferase plasmid and WT STING plasmids, or mutant mSTING constructs (Y162A, Y166A, R237A, T262A). 12 h after transfection, cells were stimulated with cGAMP, DMXAA, or telatinib. After an additional 24 h incubation, the luciferase activity was measured.

### Quantitative real-time PCR (qRT-PCR)

Total RNA was extracted from cells using Total RNA Extraction Reagent (TRIgent) (Mei5bio), and 1 μg of purified RNA was reverse-transcribed into cDNA using qPCR RT kit with gDNA remover (Mei5bio). Quantitative real-time PCR was performed using SYBR Green Mix (Mei5bio) and CFX96 (Bio-Rad). Gene expression was calculated using the ΔΔCT method and normalized to the expression of actin. The sequences of the primers were as follows: mouse Actin (5′-GCCACCAGTTCGCCATGGAT-3′; 5′-CATCACACCCTGGTGCCTAG-3′), m*Ifnb1* (5′-GCCTTTGCCATCCAAGAGATGC-3′; 5′-ACACTGTCTGCTGGTGGAGTTC-3′), m*Tnfa* (5′-GGTGCCTATGTCTCAGCCTCTT-3′, 5′-GCCATAGAACTGATGAGAGGGAG-3′), m*Isg15* (5′-CATCCTGGTGAGGAACGAAAGG-3′; 5′-CTCAGCCAGAACTGGTCTTCGT-3′), m*Cxcl10* (5′-ATCATCCCTGCGAGCCTATCCT-3′; 5′-GACCTTTTTTGGCTAAACGCTTTC-3′), m*Il6* (5′-TACCACTTCACAAGTCGGAGGC-3′;5′-CTGCAAGTGCATCATCGTTGTTC-3′), m*Ccl5* (5′-CCTGCTGCTTTGCCTACCTCTC-3′; 5′-ACACACTTGGCGGTTCCTTCGA-3′), m*Ifit3* (5′-GCTCAGGCTTACGTTGACAAGG-3′;5′-CTTTAGGCGTGTCCATCCTTCC-3′), m*Ifitm1* (5′-GCCACCACAATCAACATGCCTG-3′; 5′-ACCCACCATCTTCCTGTCCCTA-3′), human ACTIN (5′-CACCATTGGCAATGAGCGGTTC-3′; 5′-AGGTCTTTGCGGATGTCCACGT-3′), h*IFNB1* (5′- CTTGGATTCCTACAAAGAAGCAGC-3′; 5′-TCCTCCTTCTGGAACTGCTGCA-3′), h*TNFA* (5′-CTCTTCTGCCTGCTGCACTTTG-3′, 5′-ATGGGCTACAGGCTTGTCACTC-3′), h*ISG15* (5′-CTCTGAGCATCCTGGTGAGGAA-3′; 5′-AAGGTCAGCCAGAACAGGTCGT-3′), h*CXCL10* (5′-GGTGAGAAGAGATGTCTGAATCC-3′; 5′-GTCCATCCTTGGAAGCACTGCA-3′), h*IL6* (5′-AGACAGCCACTCACCTCTTCAG-3′; 5′-TTCTGCCAGTGCCTCTTTGCTG-3′), h*CCL5* (5′-CCTGCTGCTTTGCCTACATTGC-3′; 5′-ACACACTTGGCGGTTCTTTCGG-3′), h*IFIT3* (5′-CCTGGAATGCTTACGGCAAGCT-3′; 5′-GAGCATCTGAGAGTCTGCCCAA-3′), h*IFITM1* (5′-GGCTTCATAGCATTCGCCTACTC-3′; 5′-AGATGTTCAGGCACTTGGCGGT-3′).

### Western blot analysis

Cells were lysed in lysis buffer (20 mM Tris-HCl pH 7.4, 150 mM NaCl, 1% (v/v) TritonX-100, 10% (v/v) glycerol, 0.1% SDS, 1 mM EDTA, 1 mM Na_3_VO_4_, 25 mM β-glycerophosphate) supplemented with PMSF and leupeptin on ice for 30 min. The cell lysates were centrifuged at 12,000 rpm at 4 °C, then boiled at 95 °C for 10 min in loading buffer. Subsequently, proteins were separated by 12% SDS-PAGE gels and then transferred onto the PVDF membrane. The membranes were then blocked with 5% w/v nonfat dry milk for 1 h. After blocking, the membranes were incubated with primary specific antibodies overnight at 4 °C. Following washes with TBST buffer three times, membranes were incubated with secondary antibodies (HRP-Goat Anti-Rabbit IgG(H + L) (#HX2031) and HRP-Goat Anti-Mouse IgG(H+L) (#HX2032) were purchased from huaxingbio) at room temperature for 1 h. After washing three times, the detection was performed using ECL Western HRP Substrate (Mei5bio) and an Imaging System (Tanon, 5200). The band intensities were quantified using ImageJ, normalized to their corresponding loading control, and then presented as values relative to the control group within each experiment.

### ELISA

RAW, THP1, and hPBMCs were treated with telatinib for the indicated times shown in the figure legends, then supernatants were collected. The concentration of IFN-β was measured by Human or Murine IFN-beta bioluminescent ELISA kit 2.0 (Invivogen) following the manufacturer’s instructions.

### Flow cytometry

Tumors from tumor-bearing mice were harvested and cut into small pieces, then dissociated to single cells by dissociation buffer (RPMI-1640, 1 mg/ml collagenase IV, 4 U/ml DNase I) at 37 °C for 1h. After being filtered by cell strainers and centrifuged at 300 g for 5 min, cells were resuspended in red blood cell lysis buffer (Biolegend, 420,301) for 5 min. After washing with RPMI-1640, cells were stimulated with Cell Activation Cocktail (with Brefeldin A) for 4 h (Biolegend, 423303). After that, cells were incubated with antibodies: Pacific Blue anti-mouse CD45 (Biolegend, Cat# 157212; RRID: AB_2876534), PerCP/Cyanine5.5 anti-mouse CD3 (Biolegend, Cat# 100218; RRID: AB_1595492), and PE anti-mouse NK-1.1 (Biolegend, Cat# 156504; RRID: AB_2783136). Subsequently, cells were stained with Fixable Viability Dye eFluor 506 (Invitrogen, 65-0866-14), and then fixed in Fixation Buffer (Biolegend, 420801). After washing twice, cells were analyzed by BD LSRFortessa Analyzer (BD Biosciences) The results were analyzed using FlowJo.

### RNA-seq

THP1 cells were treated with or without telatinib (25 μM) for 12 h. Total RNA was extracted using TRIzol reagent (15596026, Invitrogen). RNA purity and quantification were evaluated using the Qubit4.0 (Thermo Scientific); RNA integrity was assessed using Agilent 2100 Bioanalyzer (Agilent Technologies); The libraries were constructed using Hieff NGS Ultima Dual-mode mRNA Library Prep Kit for Illumina (Cat#12301, Yeasen Biotechnology (Shanghai) Co, Ltd). RNA-seq was performed on DNBSEQ-T7 platform (Geneplus-Shenzhen). Differential expression analysis of two groups was performed using the DESeq2 R package (1.26.0). The resulting *p* values were adjusted using Benjamini and Hochberg's approach for controlling the false discovery rate. KOBAS software to test the statistical enrichment of differential expression genes in KEGG pathways.

### Surface Plasmon Resonance (SPR)

SPR experiments were performed using a Biacore 8K+ (Cytiva) at 25 °C. mSTING (154−340) was captured onto flow cells of a CM5 chip (29104988), achieving 4000−7000 response units (RU), whereas another flow cell was left empty for reference subtraction. Compounds were dissolved in 100% DMSO and diluted to working concentrations in 5% DMSO/PBST buffer, then subjected to twofold serial dilution, maintaining a DMSO final concentration of 5%. The experiments were performed using multi-cycle kinetics with a contact time of 90 s, diss time of 90 s, and flow rate of 30 μl/min. Kinetic curve fittings and K_D_ calculations were done with a 1:1 binding model and the Biacore Evaluation software.

### Protein expression and purification

The cDNA coding mouse STING (mSTING) residues 154 to 340 was cloned into the pET28b vector with an N-terminal His6-SUMO tag. For protein purification, the transformed *E.coli* BL21 (DE3) cells were cultured in LB medium (JSENB) at 37 °C at 220 rpm to an OD600 of 0.6 to 0.9 and then incubated overnight with 0.1 mM isopropyl-D-1-thiogalactopyranoside (IPTG) at 16 °C. The cells were harvested at 4200 rpm at 4 °C for 25 min. The pellet was resuspended in 50 mM Tris–HCl buffer at pH 7.5 containing 150 mM NaCl and 0.2 mM Phenylmethanesulfonyl fluoride (PMSF), followed by sonication on ice. The cell lysate was then centrifuged at 14,000 rpm for 50 min, and the supernatant was incubated with a nickel–nitrilotriacetic acid (Ni-NTA) affinity chromatography column (GE Healthcare). The 6×His tag was cleaved by SUMO protease (Ulp1) with overnight incubation at 4 °C. The protein was further purified by gel filtration chromatography using a Superdex 200 10/300 column (GE Healthcare) in 10 mM Tris pH 7.5, 100 mM NaCl. Peak fractions were collected, concentrated, and added with 5% glycerin and kept at −80 °C before use.

### Crystallization and data collection

Purified mSTING protein (4.5 mg/ml) was mixed with TTB-7 in a 1:8 M ratio. Crystals were grown at 18 °C by the hanging-drop vapor diffusion method. The crystallization conditions were 0.5 M Sodium citrate pH 5.5, 16% PEG4000. The crystals were flash-frozen in liquid nitrogen in the reservoir solution supplemented with 20% ethylene glycerol as a cryoprotectant and briefly soaked before flash. Diffraction data were collected using the Shanghai Synchrotron Radiation Facility (SSRF) and processed with the HKL2000.

### Structure determination and refinement

The structure of the mSTING bound to TTB-7 was determined by molecular replacement (MR) using the structural model of mSTING reported previously [Protein Data Bank (PDB) ID code 4JC5] as the search model using Phaser in the Phenix package. Subsequently, the initial model was subjected to manual rebuilding in Coot and refined with Phenix. Details of data quality and structure refinement are summarized in Table S1. All structural figures were generated with PyMOL (The PyMOL Molecular Graphics System, Version 2.0 Schrödinger, LLC). Structural superpositions were performed in PyMOL, and the calculation of RMSD values was based on the Cα atoms of the aligned residues.

### Statistical analysis

GraphPad Prism nine was used for data analysis and generating all charts. Statistical significance was calculated using *t* test, one-way ANOVA, or two-way ANOVA and represented as follows: ns, *p* > 0.05; ∗, *p* < 0.05; ∗∗, *p* < 0.01; ∗∗∗, *p* < 0.001; ∗∗∗∗, *p* < 0.0001. Data are shown as the mean ± SD.

## Data availability

The crystal structure of STING bound to TTB-7 is available at PDB under the accession code 9LTF. Data reported in this paper will be shared by the lead contact upon request. This paper does not report original code. Any additional information required to reanalyze the data reported in this paper is available from the lead contact upon request.

## Supporting information

This article contains supporting information (72, 73, 74, 75).

## Conflict of interest

The authors declare that they do not have any conflicts of interest with the content of this article.
