# Supplementary material for: Activation of stimulator of interferon genes (STING) and inhibition of vascular endothelial growth factor receptor (VEGFR) by telatinib induce antitumor activity[image]
Source: J Biol Chem. 2025 Dec 9;302(2):111038. doi: 10.1016/j.jbc.2025.111038 (PMC12907715; doi:10.1016/j.jbc.2025.111038)
Supplement: Supporting information [file mmc1.docx]

**Supplemental Information for**

**Activation of stimulator of interferon genes (STING) and inhibition of vascular endothelial growth factor receptor (VEGFR) by telatinib induce antitumor activity**

Yi Wang, Yanfei Hou, Jing Han, Zhengyin Zhang, Yiyang Cheng, Jiaming Yang, Luqiu Mou, Shilong Fan, Peiyuan Liu, Kehong Chen, Yuanwei Dai, and Conggang Zhang

**This PDF file includes:**

Figures S1 to S6

Table S1

Chemical synthesis

SI References


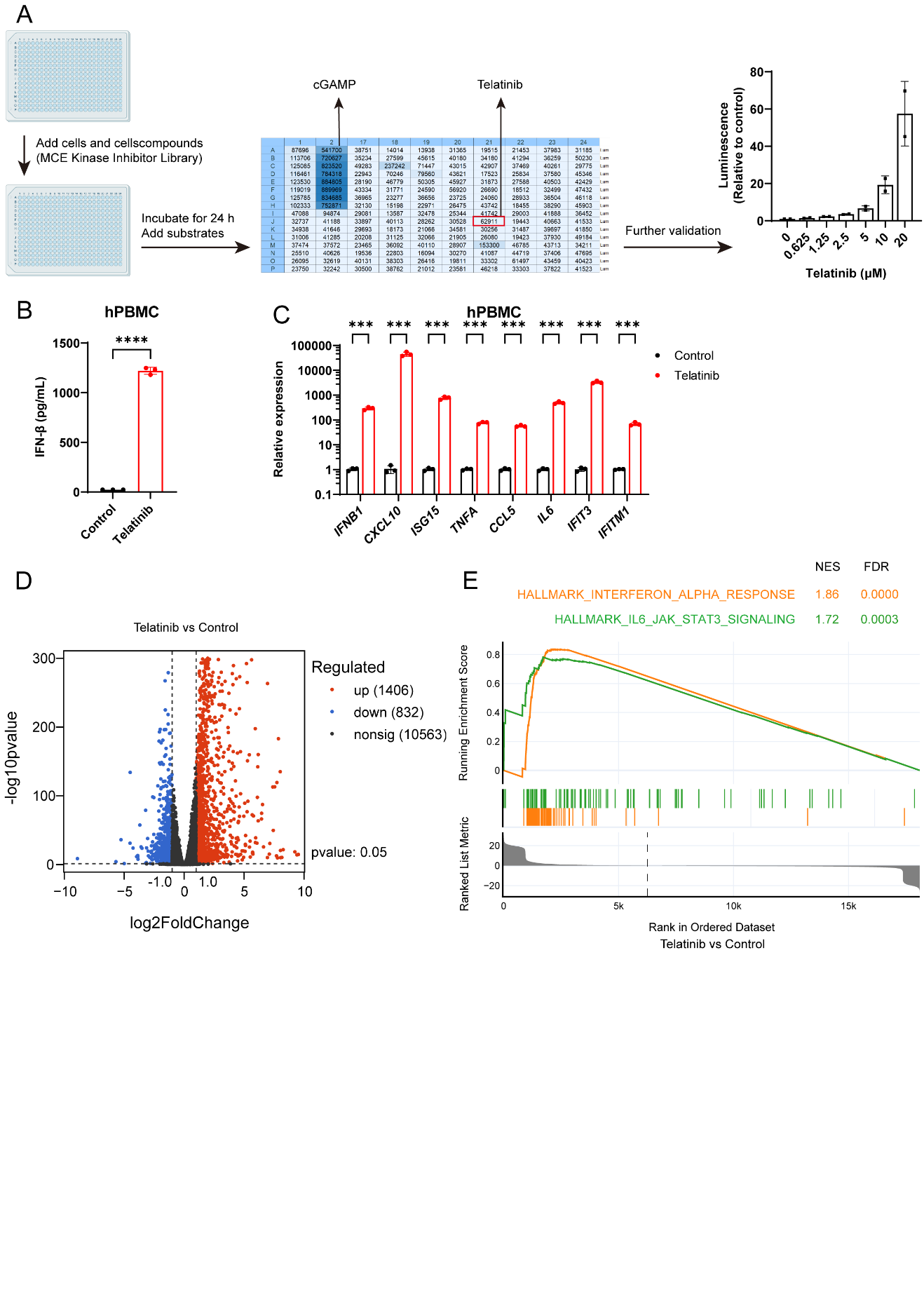


**Figure S1.** **Telatinib activates the type I interferon responses, related to Figure 1.** (A) The workflow of high-throughput screening. The luminescence data are normalized to the control group. (B) IFN-β production in hPBMCs stimulated with telatinib (25 μM) for 12 h. (C) q-PCR analysis of target gene expression in hPBMC treated as (B) (n = 3 biological replicates). (D and E) RNA-seq analysis of THP1-Lucia ISG cells incubated with or without telatinib (25 μM) for 12 h. (D) Volcano plots showing changes in gene expression by the telatinib-treated group versus the control group. (E) GSEA of RNA-seq data showing enrichment of signaling. ****P* < 0.001; *****P* < 0.0001. Data are shown as the mean ± SD. Statistical significance was determined using an unpaired *t*-test.


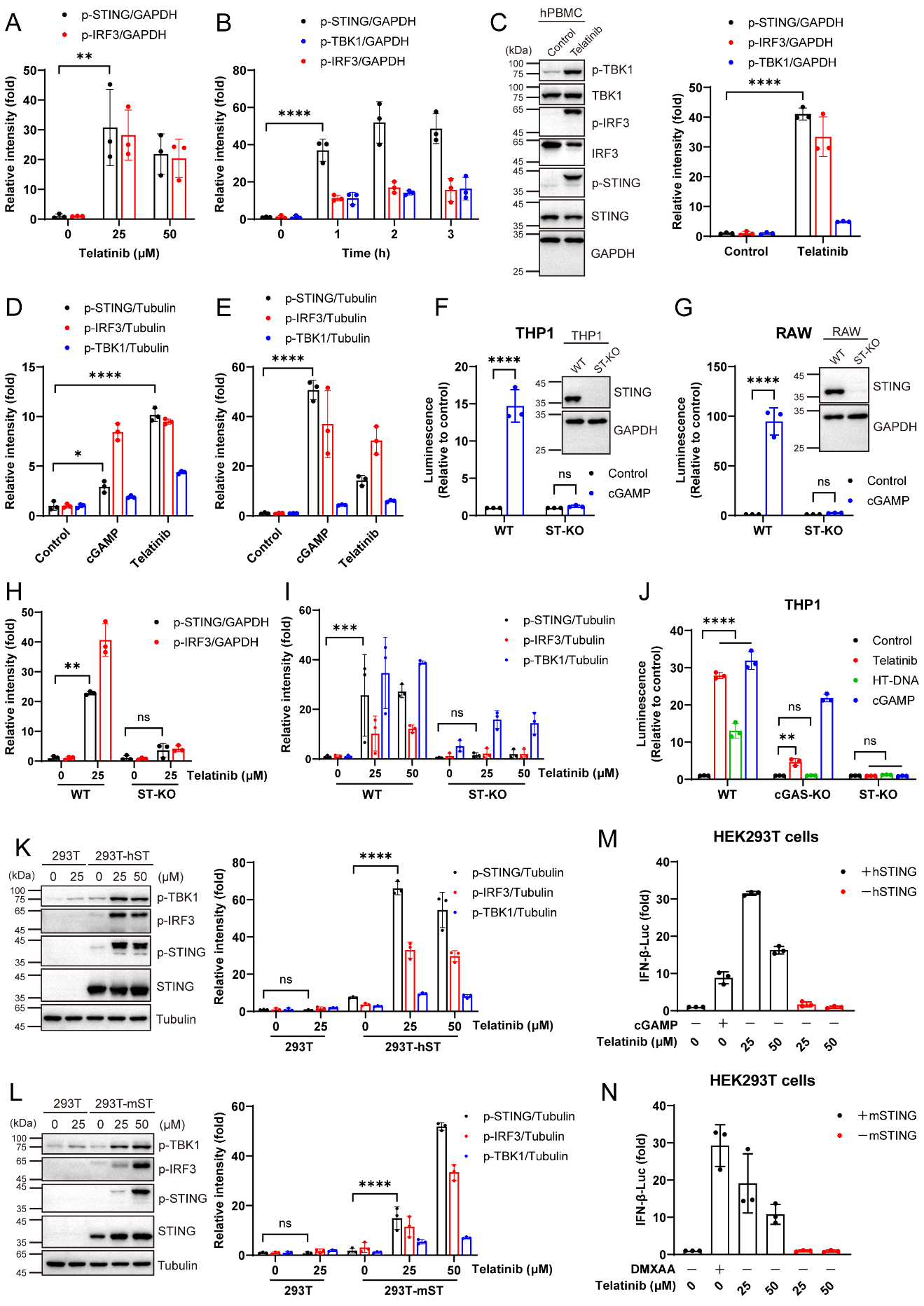


**Figure S2.** **Telatinib activates the cGAS-STING pathway in a STING-dependent manner, related to Figure 2.** (A) Quantification of the western blot results in Figure 2A, n = 3 biological replicates. (B) Quantification of the western blot results in Figure 2B, n = 3 biological replicates. (C) Left: Western blot analysis of hPBMC treated with or without telatinib (25 μM) for 2 h. Right: Western blotting quantification was performed using ImageJ (n = 3 biological replicates). (D) Quantification of the western blot results in Figure 2C, n = 3 biological replicates. (E) Quantification of the western blot results in Figure 2D, n = 3 biological replicates. (F and G) Identification of STING knockout in THP1-Lucia ISG cells (F) and RAW-Lucia ISG cells (G) by luciferase activity and western blot. The luminescence data are normalized to the control group. (H) Quantification of the western blot results in Figure 2E, n = 3 biological replicates. (I) Quantification of the western blot results in Figure 2F, n = 3 biological replicates. (J) Luciferase activity of THP1-Lucia ISG cells (WT, cGAS-KO, and ST-KO) treated with telatinib (25 μM), cGAMP (2 μM), and HT-DNA (1μg/mL) for 24 h. The luminescence data are normalized to the control group. (K and L) Left: Western blot analysis of WT HEK293T cells and HEK293T cells stably expressing hSTING (K) or mSTING (L) treated with telatinib at the indicated concentrations. Right: Western blotting quantification was performed using ImageJ (n = 3 biological replicates). (M and N) HEK293T cells were transfected with IFN-β plasmids with or without hSTING (M) or mSTING (N) plasmids, then incubated with telatinib for 18 h, followed by measurement of luciferase activity. The luminescence data are normalized to the control group. All data are representative of three independent experiments. ***P* < 0.01; ****P* < 0.001; *****P* < 0.0001; NS, not significant (*P* > 0.05). Data are shown as the mean ± SD. Statistical significance was determined using two-way ANOVA.


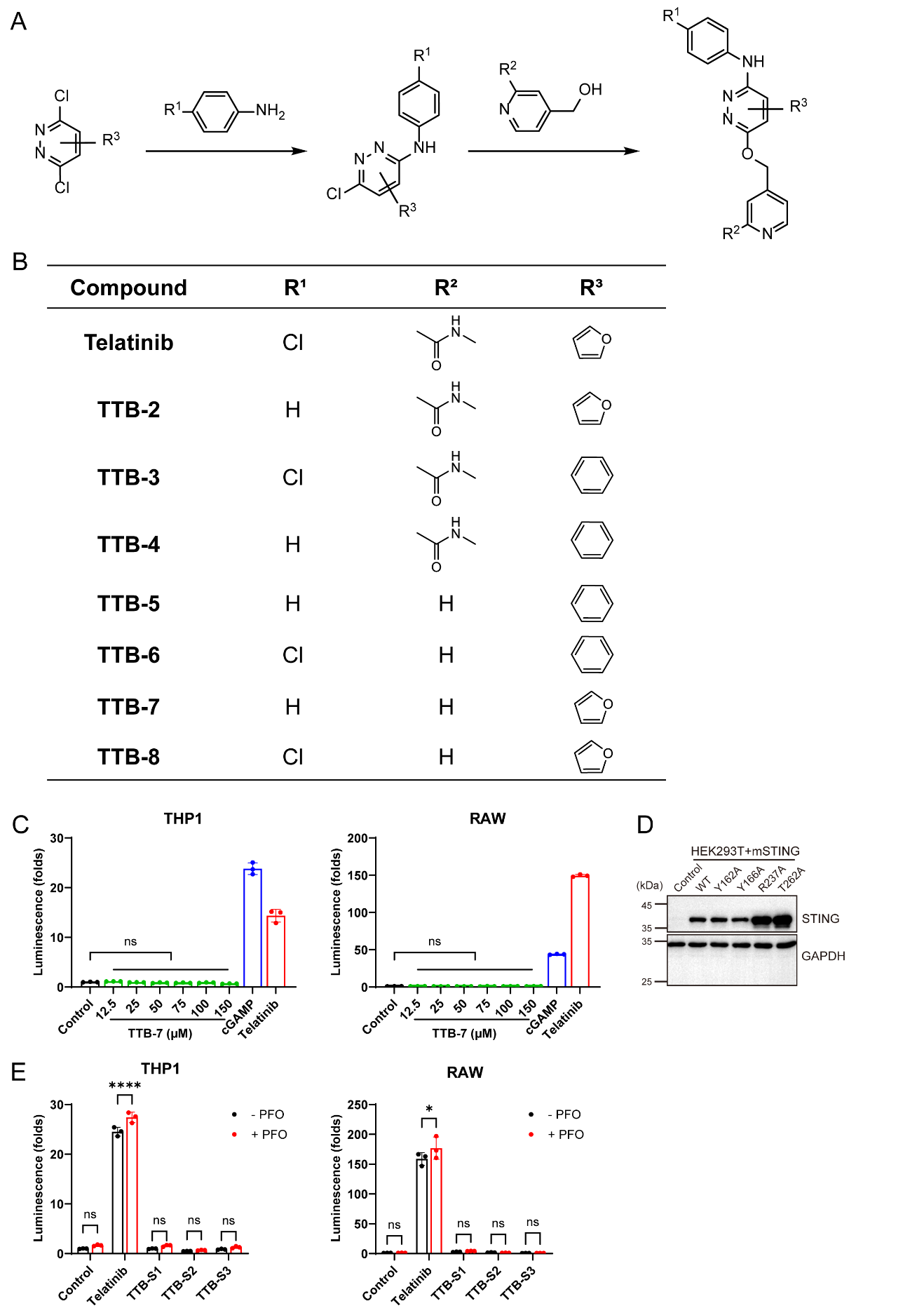


**Figure S3.** **Structure and cellular activity of telatinib analogs, related to Figure 3.** (A) Synthesis route of telatinib analogs. Details are shown in the supporting information of the chemical synthesis. (B) Chemical structure of telatinib and its analogs. (C) Luciferase activity of THP1-Lucia ISG and RAW-Lucia ISG cells treated with cGAMP (2 μM), telatinib (25 μM), and increasing doses of TTB-7 for 24 h. The luminescence data are normalized to the control group. (D) STING expression in HEK293T cells transiently transfected with WT or mutated mSTING plasmids. (E) Luciferase activity of THP1-Lucia ISG and RAW-Lucia ISG cells treated with telatinib (25 μM), TTB-S1 (25 μM), TTB-S2 (25 μM), and TTB-S3 (25 μM) in the presence or absence of PFO for 24 h. The luminescence data are normalized to the control group. **P* < 0.05; *****P* < 0.0001; NS, not significant (*P* > 0.05). Data are shown as the mean ± SD. Statistical significance was determined using one-way ANOVA (C) or two-way ANOVA (D).


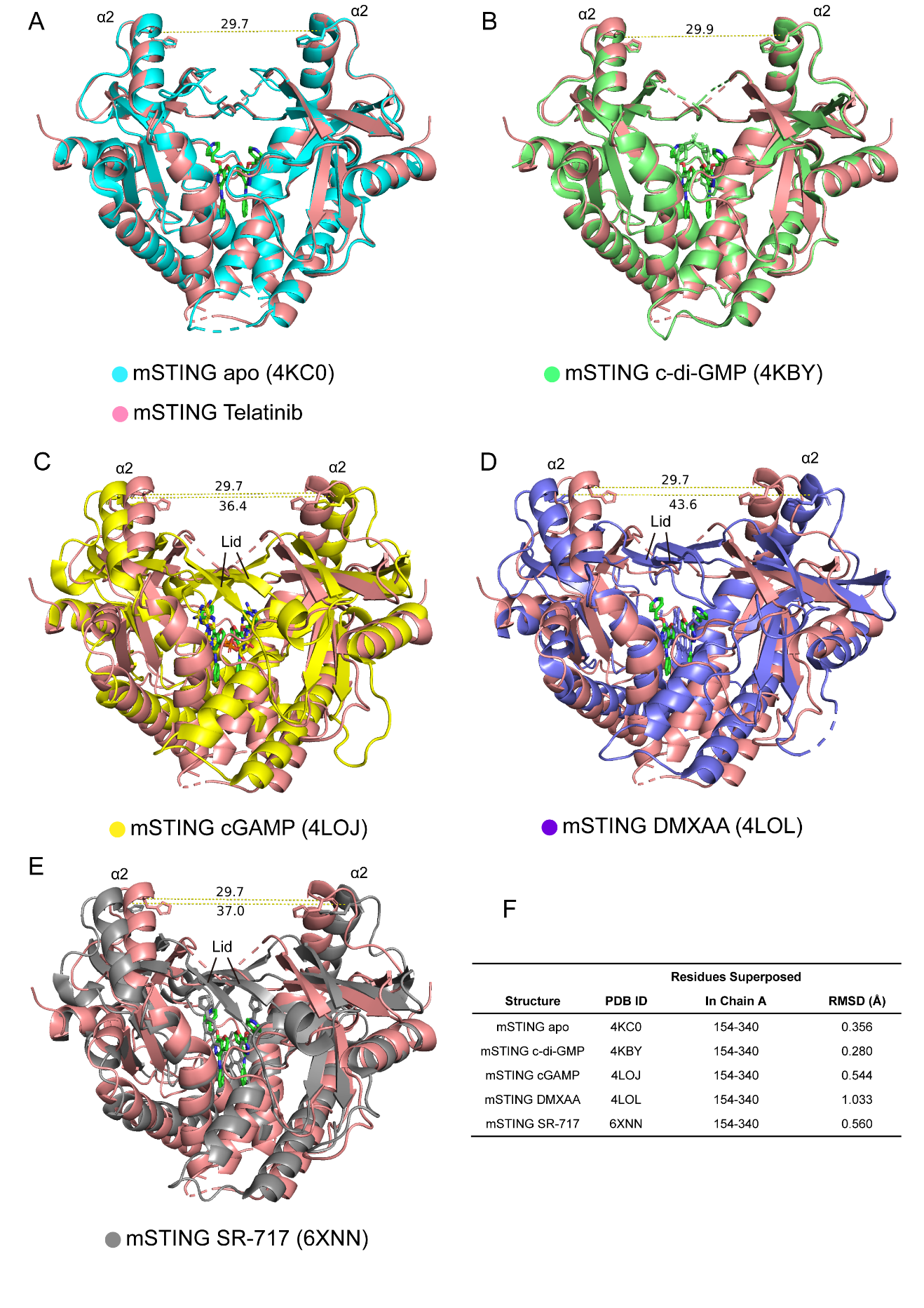


**Figure S4.** **Superposition of co-crystal structures, related to Figure 3.** (A) Superposition of the TTB-7-bound structure of mSTING (red) with the unbound (apo) structure of mSTING (blue) (PDB ID 4KC0). (B) Superposition of the TTB-7-bound structure of mSTING (red) with the c-di-GMP-bound structure of mSTING (green) (PDB ID 4KBY). (C) Superposition of the TTB-7-bound structure of mSTING (red) with the cGAMP-bound structure of mSTING (yellow) (PDB ID 4LOJ). (D) Superposition of the TTB-7-bound structure of mSTING (red) with the DMXAA-bound structure of mSTING (purple) (PDB ID 4LOL). (E) Superposition of the TTB-7-bound structure of mSTING (red) with the DMXAA-bound structure of mSTING (grey) (PDB ID 6XNN). (F) Regions of the protein used for the superpositions and the corresponding RMSDs. Residues 154-340 of the TTB-7-bound mSTING structure were used for the superpositions with the corresponding residues from a single chain of other STING structures.


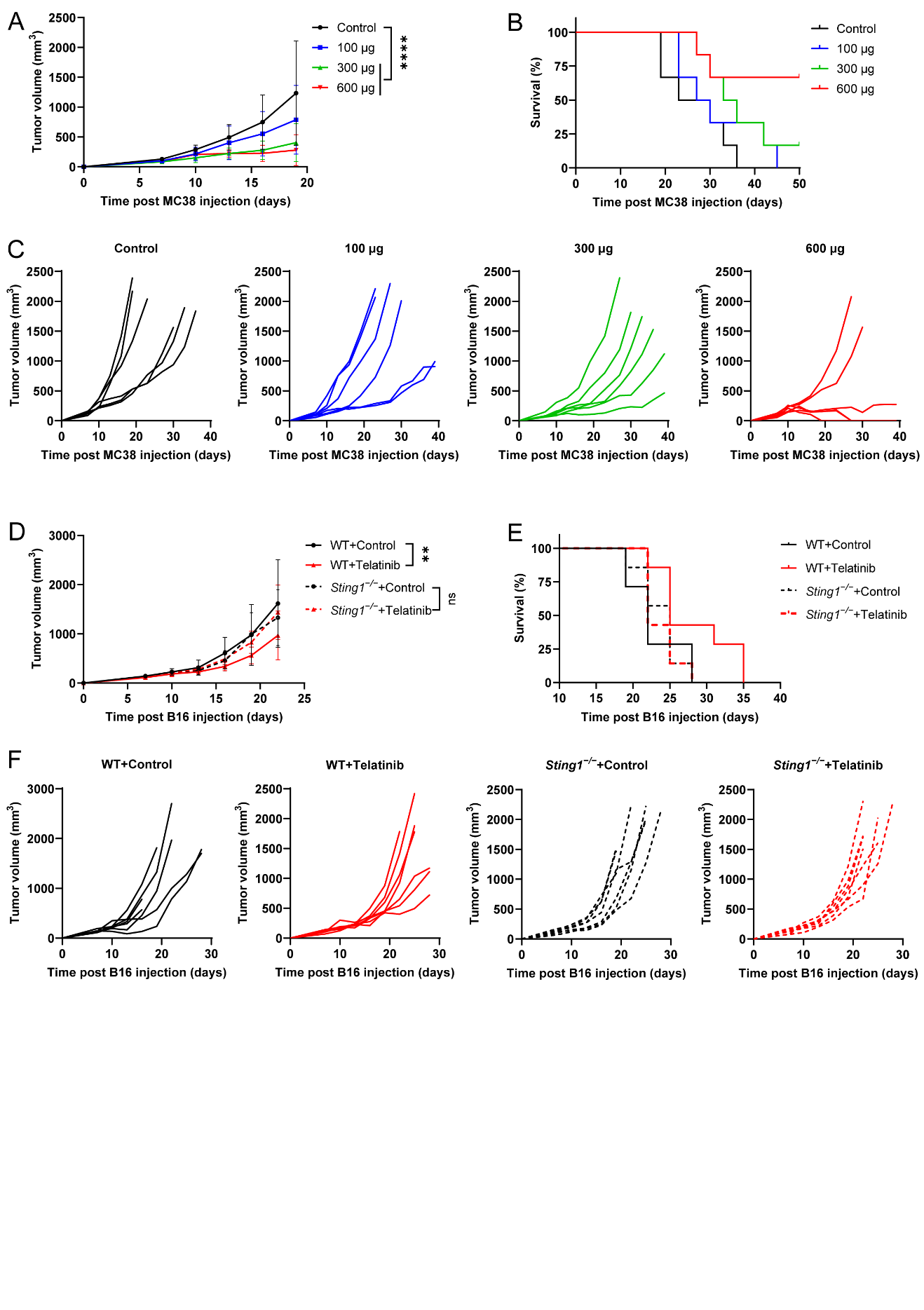


**Figure S5.** **Therapeutic activity of telatinib, related to Figure 5.** (A-C) WT MC38 tumor-bearing mice (n = 6) were treated with telatinib by oral administration at different doses (100, 300, 600 μg) on day 7, 10, 13, and 16. Tumor growth curve (A), survival curve (B), and individual tumor growth (C) of WT MC38 tumor-bearing mice are shown. (D-F) WT or *Sting1^−/−^* B16 tumor-bearing mice (n = 7) were treated with telatinib by intratumoral (IT) administration (600 μg) on day 7, 10, 13, and 16. Tumor growth curve (D), survival curve (E), and individual tumor growth (F) of B16 tumor-bearing mice are shown. ***P* < 0.01; *****P* < 0.0001; NS, not significant (*P* > 0.05). Data are shown as the mean ± SD. Statistical significance was determined using two-way ANOVA.


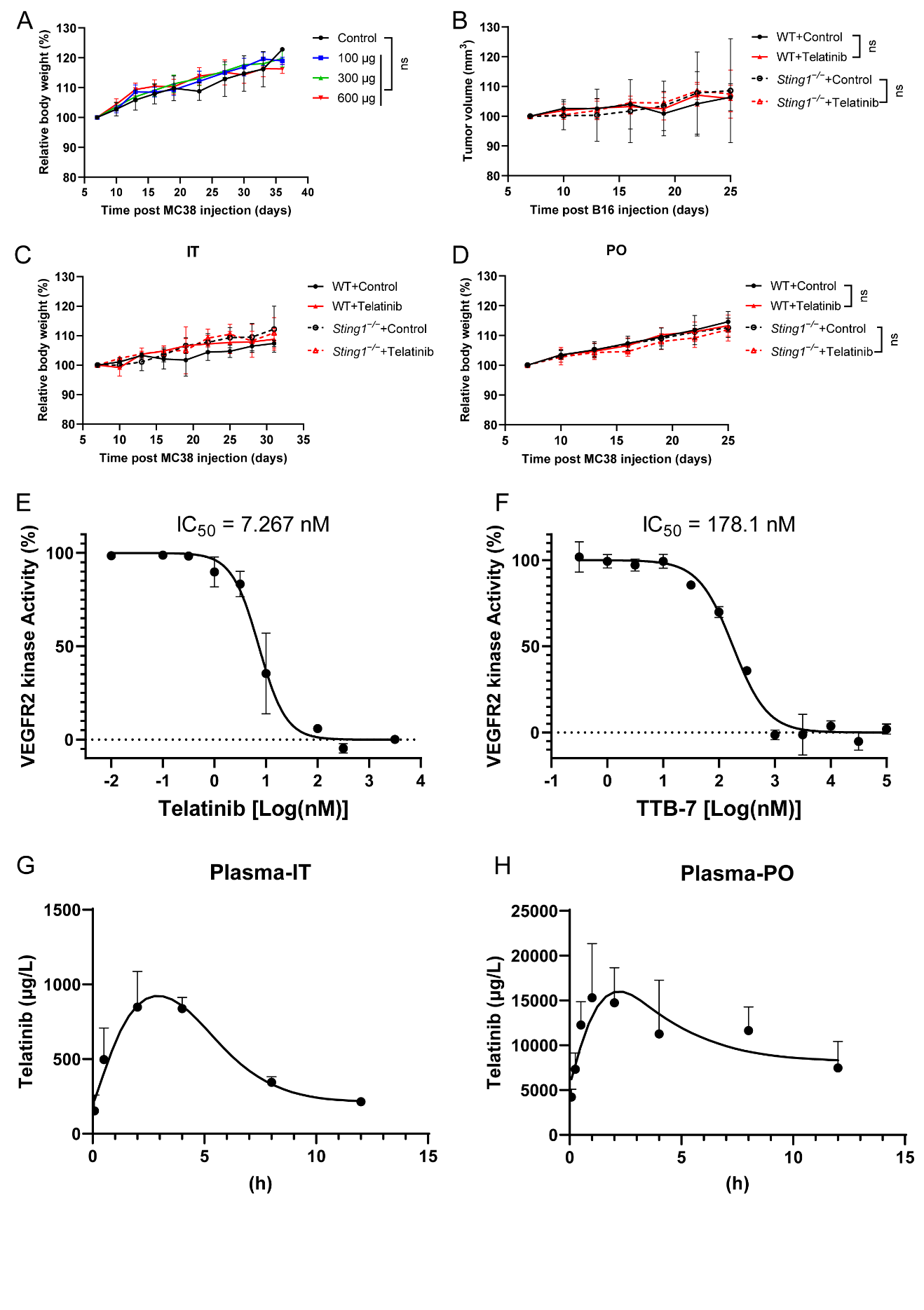


**Figure S6.** **Mouse body weight changes and characteristics of telatinib, related to Figure 5.** (A-D) Body weight of mice treated as described in Figure S5A (A), Figure S5D (B), Figure 5A (C), and Figure 5H (D). (E and F) Potency of telatinib (E) and TTB-7 (F) in inhibiting VEGFR2 kinase activity measured by ELISA kit. (G and H) Pharmacokinetic properties of telatinib administered intratumorally (15 mg/kg) (G) or orally (30 mg/kg) (H) in healthy WT C57BL/6 mice.

**Table S1.** **X-ray data and refinement statistics, related to Figure 3.**

| PDB code | 9LTF |
| --- | --- |
| Wavelength (Å) | 0.9789 |
| Resolution range (Å) | 34.47 – 1.92(1.95-1.92) |
| Space group | P 3121 |
| Unit cell (Å, °) | 79.61 79.61 49.91 90 90 120 |
| Unique reflections | 14233 |
| Completeness (%) | 99.9 (96.67) |
| Redundancy | 18.8 (19.4) |
| Mean I/sigma(I) | 33.17 (2.63) |
| Wilson B-factor(Å^2^) | 31.0 |
| R-merge | 0.14 (0.981) |
| CC1/2 | 0.997 (0.86) |
| Reflections used in the refinement | 14093 (1363) |
| Reflections used for R-free | 745 (82) |
| R-work | 0.1973 |
| R-free | 0.2385 |
| Number of non-hydrogen atoms | 1532 |
| macromolecules | 1444 |
| ligands | 24 |
| RMS (bonds) | 0.028 |
| RMS (angles) | 1.12 |
| Ramachandran favored (%) | 97.08 |
| Ramachandran allowed (%) | 2.92 |
| Ramachandran outliers (%) | 0.00 |
| Rotamer outliers (%) | 0.62 |
| Clashscore | 6.22 |
| Average B-factor | 34.05 |
| macromolecules | 33.86 |
| ligands | 33.51 |
| solvent | 38.65 |

**Chemical synthesis**

**General Information**

All commercially purchased materials are used directly in accordance with the marked purity. Stirring and heating are carried out in Heidolph magnetic stirrers. ^1^H NMR and ^13^C NMR spectra were recorded on Bruker NMR (400 MHz) or Agilent 600MR DD2 (600 MHz) spectrometer at room temperature. ^1^H NMR chemical shifts were recorded relative to the solvent residual peak (CDCl_3_ at 7.26 ppm, DMSO-*d*_6_ at 2.50 ppm). ^13^C NMR chemical shifts are reported relative to the solvent residual peak (CDCl_3_ at 77.0 ppm, DMSO-*d*_6_ 39.7 at ppm). Chemical shifts (δ) are reported in ppm, and coupling constants (*J*) are in Hertz (Hz). The following abbreviations were used to explain the multiplicities: s = singlet, d = doublet, t = triplet, q = quartet, m = multiplet, sept = septet. All deuterated solvents were purchased from Cambridge Isotope Laboratories. High resolution mass spectra (HR-MS) were acquired on an Agilent 65467 Q-TOF MS (Agilent, USA) with an ESI source. TLCs are carried out on pre-coated GF254 silica plates (Yantai Huayang Co., Ltd) with visualization via 254/365 nm UV light. Flash chromatography uses silica gel powder (200-300 mesh, Shanghai yuanye Bio-Technology Co., Ltd). All anhydrous solvents are purified by the FLEANO solvent purification system.

**Synthetic procedures**

**4-(Hydroxymethyl)-N-methylpicolinamide (2)**

To a solution of compound **1** (1.0 g, 6.5 mmol) in DMF (8.0 mL) were added methylamine hydrochloride (661.7 mg, 9.8 mmol), HATU (3.7 g, 9.8 mmol) and DIPEA (3.4 mL, 19.6 mmol). The reaction was stirred at room temperature for 5 h. After full conversion, the reaction was diluted with EtOAc (20.0 mL×3) and washed with brine (20.0 mL×3), dried with anhydrous Na_2_SO_4_. Next, the solvent was evaporated under reduced pressure, and the residue was purified by silica gel column chromatography (eluting with dichloromethane/methanol=10/l) to afford **2** (972.2 mg, 90% yield) as a yellow oil. **^1^H NMR (400 MHz, DMSO-*d*_6_)** δ 8.75 (q, J = 5.3 Hz, 1H), 8.54 (d, J = 5.0 Hz, 1H), 8.01 (s, 1H), 7.49 (dd, J = 5.0, 1.7 Hz, 1H), 4.62 (s, 2H), 2.82 (d, J = 4.9 Hz, 3H). The NMR data is consistent with the reported(1).

**4,7-Dichlorofuro[2,3-d]pyridazine (4)**

To a solution of compound **3** (500.0 mg, 2.7 mmol) in EtOH (5.0 mL) was added hydrazine hydrate (55% N_2_H_4_, 0.5 mL, 16.3 mmol). After addition, the reaction mixture was heated to reflux for 5.5 h, then, solids were slowly precipitated during cooling to room temperature. The volatiles were removed under reduced pressure to furnish a white paste which was suspended in water and filtered. The white solid was washed with water. The solid was suspended in aqueous 2N HCl (5.0 mL), and the mixture was refluxed for 4 h, the white slurry was cooled to room temperature and filtered. The solid was washed thoroughly with water and dried under vacuum to get a white solid. Next, the solid was dissolved in pyridine (5.0 mL), POCl_3_ (168.0 mL, 18.1 mmol) was added in the mixture. Then, the reaction was refluxed for 8 h. After full conversion, the reaction was diluted with DCM (20.0 mL×3) and washed with brine (20.0 mL×3), dried with anhydrous Na_2_SO_4_. The solvent was evaporated under reduced pressure to afford **4** (332.3 mg, 65%) as an orange-red solid. **^1^H NMR (400 MHz, DMSO-*d*_6_)** δ 8.64 (d, J = 2.0 Hz, 1H), 7.41 (d, J = 2.0 Hz, 1H). The NMR data is consistent with the reported(2).

**7-Chloro-N-phenylfuro[2,3-d]pyridazin-4-amine (5)**

Compound **4** (300.0 mg, 1.6 mmol) was dissolved in EtOH (15.0 mL). Aniline (158.2 mg, 1.7 mmol) was added to the solution. Then, the reaction was refluxed for 4 h. After full conversion, the solvent was evaporated under reduced pressure, and the residue was purified by silica gel column chromatography (eluting with petroleum ether/dichloromethane =1/1) to afford **5** (215.6 mg, 55%) as a yellow powder(3). **^1^H NMR (400 MHz, CDCl_3_)** δ 7.69 (d, J = 2.2 Hz, 1H), 7.42 (ddd, J = 7.2, 6.2, 2.6 Hz, 2H), 7.39 – 7.35 (m, 2H), 7.23 – 7.17 (m, 1H), 6.34 (d, J = 2.2 Hz, 1H). The NMR data is consistent with the reported^3^.

**N-Methyl-4-(((4-(phenylamino)furo[2,3-d]pyridazin-7-yl)oxy)methyl) picolinamide (TTB-2)**

To a solution of compound **5** (300.0 mg, 1.2 mmol) in toluene (10.0 mL) were added KOH (140.3 mg, 2.5 mmol), compound **2** (415.2 mg, 2.5 mmol) and 18-crown-6 (13.2 mg, 0.05 mmol). After addition, the reaction was stirred at 85°C for 12 h. Then, the solvent was evaporated under reduced pressure, and the residue was purified by silica gel column chromatography (eluting with petroleum ether/ ethyl acetate =1/1) to afford **TTB-2** (225.1 mg, 50%) as a white solid. **^1^H NMR (400 MHz, CDCl_3_)** δ 8.52 (d, J = 5.0 Hz, 1H), 8.32 (d, J = 1.8 Hz, 1H), 8.05 (s, 1H), 7.68 (d, J = 2.1 Hz, 1H), 7.56 (dd, J = 4.9, 1.7 Hz, 1H), 7.49 – 7.44 (m, 2H), 7.31 (t, J = 7.8 Hz, 2H), 7.10 – 7.05 (m, 1H), 6.57 (d, J = 2.1 Hz, 1H), 5.73 (s, 2H), 3.03 (d, J = 5.2 Hz, 3H).**^13^C NMR (100 MHz, CDCl_3_)** δ 165.03, 151.37, 150.34, 149.42, 148.42, 147.75, 146.64, 143.82, 140.13, 129.25, 124.38, 123.52, 120.95, 120.37, 119.27, 119.14, 105.17, 66.54, 26.29. **HRMS** (ESI/TOF) m/z: calculated for C_20_H_18_N_5_O_3_ [M+H]^+^ 376.1410 found 376.1402.

**4-Chloro-N-(4-chlorophenyl)phthalazin-1-amine (7)**

To a solution of **6** (500.0 mg, 2.5 mmol) in EtOH (30.0 mL) was added 4-chloroaniline (343.0 mg, 2.7 mmol). Then, the reaction mixture was refluxed for 0.5 h. Next, the mixture was cooled to room temperature, filtered, and washed with petroleum ether. The filter cake was purified by silica gel column chromatography (eluting with petroleum ether/ ethyl acetate =1/1) to afford **7** (686.4 mg, 95%) as a yellow solid. **^1^H NMR (400 MHz, DMSO-*d*_6_)** δ 10.01 (s, 1H), 8.79 – 8.72 (m, 1H), 8.21 (dt, J = 8.0, 2.9 Hz, 1H), 8.17 – 8.12 (m, 2H), 7.91 – 7.83 (m, 2H), 7.54 – 7.43 (m, 2H). The NMR data is consistent with the reported(4).

**4-(((4-((4-Chlorophenyl)amino)phthalazin-1-yl)oxy)methyl)-N-methylpicolinamide (TTB-3)**

To a solution of compound **7** (289.0 mg, 1.0 mmol) in DBU (10.0 mL) was added compound **2** (830.4 mg, 5.0 mmol) in one portion. After refluxing for 12 h, the reaction mixture was cooled to room temperature, diluted with DCM (20.0 mL×3) and washed with brine (20.0 mL×3), dried with anhydrous Na_2_SO_4_. The solvent was evaporated under reduced pressure, and the residue was purified by silica gel column chromatography (eluting with petroleum ether/ ethyl acetate =1/1) to afford **TTB-3** (230.5 mg, 55%) as a white solid. **^1^H NMR (400 MHz, CDCl_3_)** δ 8.55 (d, J = 5.0 Hz, 1H), 8.37 (s, 1H), 8.30 (s, 1H), 8.06 (s, 1H), 7.93 (s, 1H), 7.86 (dd, J = 6.1, 3.4 Hz, 2H), 7.63 (s, 2H), 7.54 (d, J = 5.0 Hz, 1H), 7.29 (d, J = 8.6 Hz, 2H), 6.90 (s, 1H), 5.72 (s, 2H), 3.05 (d, J = 5.1 Hz, 3H).**^13^C NMR (150 MHz, CDCl_3_)** δ 165.08, 150.41, 148.39, 148.13, 132.42, 132.09, 129.10, 124.29, 124.08, 121.35, 120.20, 66.56, 26.30. **HRMS** (ESI/TOF) m/z: calculated for C_22_H_19_ClN_5_O_2_ [M+H]^+^ 420.1227 found 420.1217.

**4-Chloro-N-phenylphthalazin-1-amine (8)**

To a solution of **6** (500.0 mg, 2.5 mmol) in EtOH (30.0 mL) was added aniline (251.3 mg, 2.7 mmol). Then, the reaction mixture was refluxed for 0.5 h. Next, the mixture was cooled to room temperature, filtered, and washed with petroleum ether. The filter cake was purified by silica gel column chromatography (eluting with petroleum ether/ ethyl acetate =1/1) to afford **8** (619.8 mg, 90%) as a white solid. **^1^H NMR (400 MHz, DMSO-*d*_6_)** δ 9.35 (s, 1H), 8.71 – 8.64 (m, 1H), 8.20 – 8.15 (m, 1H), 8.15 – 8.06 (m, 2H), 7.87 (d, J = 8.0 Hz, 2H), 7.38 (t, J = 7.9 Hz, 2H), 7.08 (t, J = 7.3 Hz, 1H). The NMR data is consistent with the reported(5).

**N-Methyl-4-(((4-(phenylamino)phthalazin-1-yl)oxy)methyl)picolinamide (TTB-4)**

To a solution of compound **8** (300.0 mg, 1.2 mmol) in DBU (10.0 mL), was added **2** (996.4 mg, 6.0 mmol) in one portion. After refluxing for 12 h, the reaction mixture was cooled to room temperature, diluted with DCM (20.0 mL×3) and washed with brine (20.0 mL×3), dried with anhydrous Na_2_SO_4_. The solvent was evaporated under reduced pressure, and the residue was purified by silica gel column chromatography (eluting with petroleum ether/ ethyl acetate =1/1) to afford **TTB-4** (240.3 mg, 52%) as a yellow solid. **^1^H NMR (400 MHz, CDCl_3_)** δ 8.54 (d, J = 5.0 Hz, 1H), 8.37 (s, 1H), 8.32 – 8.26 (m, 1H), 8.05 (s, 1H), 7.94 (d, J = 4.8 Hz, 1H), 7.86 (dt, J = 6.2, 3.8 Hz, 2H), 7.63 (d, J = 8.0 Hz, 2H), 7.55 (d, J = 5.0 Hz, 1H), 7.34 (t, J = 7.7 Hz, 2H), 7.04 (t, J = 7.4 Hz, 1H), 5.72 (s, 2H), 3.04 (d, J = 5.1 Hz, 3H).**^13^C NMR (100 MHz, CDCl_3_)** δ 165.08, 150.38, 148.36, 148.23, 132.28, 131.95, 129.18, 124.31, 123.97, 122.79, 121.39, 120.22, 120.16, 66.51, 26.29. **HRMS** (ESI/TOF) m/z: calculated for C_22_H_20_N_5_O_2_ [M+H]^+^ 386.1617 found 386.1612.

**N-Phenyl-4-(pyridin-4-ylmethoxy)phthalazin-1-amine (TTB-5)**

To a solution of compound **9** (300.0 mg, 1.2 mmol) in DBU (10.0 mL) was added 4-pyridinecarbinol (654.3 mg, 6.0 mmol). After refluxing for 12 h, the reaction mixture was cooled to room temperature, diluted with DCM (20.0 mL×3) and washed with brine (20.0 mL×3), dried with anhydrous Na_2_SO_4_. The solvent was evaporated under reduced pressure, and the residue was purified by silica gel column chromatography (eluting with petroleum ether/ ethyl acetate =1/1) to afford **TTB-5** (326.8 mg, 83%) as a yellow solid. **^1^H NMR (400 MHz, CDCl_3_)** δ 8.66 – 8.60 (m, 2H), 8.33 – 8.24 (m, 1H), 8.02 – 7.93 (m, 1H), 7.92 – 7.83 (m, 2H), 7.66 – 7.60 (m, 2H), 7.44 (d, J = 5.5 Hz, 2H), 7.34 (t, J = 7.9 Hz, 2H), 7.05 (t, J = 7.4 Hz, 1H), 5.67 (s, 2H), 5.29 (s, 1H).**^13^C NMR (100 MHz, CDCl_3_)** δ 149.88, 146.15, 132.14, 131.80, 129.06, 123.71, 122.74, 121.86, 121.42, 120.05, 66.57. **HRMS** (ESI/TOF) m/z: calculated for C_20_H_17_N_4_O [M+H]^+^ 329.1402 found 329.1400.

**N-(4-Chlorophenyl)-4-(pyridin-4-ylmethoxy)phthalazin-1-amine (TTB-6)**

To a solution of compound **7** (289.0 mg, 1.0 mmol) in DBU (10.0 mL) was added 4-pyridinecarbinol (545.3 g, 5.0 mmol). After refluxing for 12 h, the reaction mixture was cooled to room temperature, diluted with DCM (20.0 mL×3) and washed with brine (20.0 mL×3), dried with anhydrous Na_2_SO_4_. The solvent was evaporated under reduced pressure, and the residue was purified by silica gel column chromatography (eluting with petroleum ether/ ethyl acetate =1/1) to afford **TTB-6** (307.8 mg, 85%) as a white solid(2). **^1^H NMR (400 MHz, CDCl_3_)** δ 8.61 (d, J = 5.6 Hz, 2H), 8.33 – 8.24 (m, 1H), 8.06 – 7.99 (m, 1H), 7.94 – 7.83 (m, 2H), 7.63 – 7.56 (m, 2H), 7.43 (d, J = 5.0 Hz, 2H), 7.25 (d, J = 8.6 Hz, 2H), 5.65 (s, 2H), 5.29 (s, 1H).**^13^C NMR (100 MHz, CDCl_3_)** δ 149.88, 146.09, 132.23, 131.88, 128.93, 127.39, 123.79, 121.86, 121.20, 66.59. **HRMS** (ESI/TOF) m/z: calculated for C_20_H_17_ClN_4_O [M+H]^+^ 363.1013 found 363.1016.

**N-Phenyl-7-(pyridin-4-ylmethoxy)furo[2,3-d]pyridazin-4-amine (TTB-7)**

To a solution of compound **5** (300.0 mg, 1.2 mmol) in toluene (10.0 mL) were added KOH (140.3 mg, 2.5 mmol), 4-pyridinecarbinol (272.6 mg, 2.5 mmol) and 18-crown-6 (13.2 mg, 0.05 mmol). After addition, the reaction was stirred at 85 °C for 12 h. Then, the reaction mixture was cooled to room temperature. Next, the solvent was evaporated under reduced pressure, and the residue was purified by silica gel column chromatography (eluting with petroleum ether/ ethyl acetate =1/1) to afford **TTB-7** (248.1 mg, 65%) as a white solid. **^1^H NMR (400 MHz, CDCl_3_)** δ 8.64 – 8.58 (m, 2H), 7.70 (d, J = 2.1 Hz, 1H), 7.49 – 7.40 (m, 4H), 7.32 (t, J = 7.7 Hz, 2H), 7.09 (t, J = 7.4 Hz, 1H), 6.55 (d, J = 2.0 Hz, 1H), 5.69 (s, 2H).**^13^C NMR (100 MHz, CDCl_3_)** δ 151.19, 149.86, 149.48, 146.44, 145.76, 143.77, 140.02, 129.15, 123.44, 122.08, 120.81, 118.96, 105.04, 66.60. **HRMS** (ESI/TOF) m/z: calculated for C_18_H_15_N_4_O_2_ [M+H]^+^ 319.1195 found 319.1195.

**7-Chloro-N-(4-chlorophenyl)furo[2,3-d]pyridazin-4-amine (9)**

To a solution of compound **4** (300.0 mg, 1.6 mmol) in EtOH (15.0 mL) was added 4-chloroaniline (228.6 mg, 1.8 mmol). Then, the reaction was refluxed for 4 h. After full conversion, the solvent was evaporated under reduced pressure, and the residue was purified by silica gel column chromatography (eluting with petroleum ether/ dichloromethane =1/1) to afford **9** (276.8 mg, 62%) as a yellow powder. **^1^H NMR (600 MHz, CDCl_3_)** δ 8.90 (s, 1H), 7.62 (d, J = 2.1 Hz, 1H), 7.61 (d, J = 8.7 Hz, 2H), 7.13 (d, J = 2.0 Hz, 1H), 7.04 (d, J = 8.7 Hz, 2H). The NMR data is consistent with the reported(3).

**N-(4-Chlorophenyl)-7-(pyridin-4-ylmethoxy)furo[2,3-d]pyridazin-4-amine (TTB-8)**

To a solution of compound **9** (279.0 mg, 1.0 mmol) in toluene (10.0 mL) were added KOH (117.8 mg, 2.1 mmol), 4-pyridinecarbinol (229.0 mg, 2.1 mmol) and 18-crown-6 (10.5 mg, 0.04 mmol). Then, the reaction was stirred at 85°C for 12 h. Next, the reaction was cooled to room temperature, the solvent was evaporated under reduced pressure, and the residue was purified by silica gel column chromatography (eluting with petroleum ether/ ethyl acetate =1/1) to afford **TTB-8** (216.5 mg, 62%) as a white solid. **^1^H NMR (400 MHz, CDCl_3_)** δ 8.66 – 8.60 (m, 2H), 7.77 (d, J = 2.1 Hz, 1H), 7.46 (dd, J = 10.9, 6.8 Hz, 4H), 7.30 (d, J = 8.6 Hz, 2H), 6.62 (d, J = 2.1 Hz, 1H), 5.70 (s, 2H).**^13^C NMR (150 MHz, CDCl_3_)** δ 150.66, 150.14, 149.81, 147.02, 145.66, 143.93, 138.64, 129.32, 128.44, 122.18, 121.79, 119.05, 104.62, 66.87. **HRMS** (ESI/TOF) m/z: calculated for C_18_H_14_ClN_4_O_2_ [M+H]^+^ 353.0805 found 353.0800.

**4-(((4-((4-chlorophenyl)amino)thieno[2,3-d]pyridazin-7-yl)oxy)methyl)-N-methylpicolinamide (TTB-S1)**

Compound **11** was synthesized in a similar way to compound **9**. Compound **2** (415 mg, 2.5 mmol) and compound **11** (148 mg, 0.5 mmol) were dissolved in toluene (10 mL). KOH (200 mg, 5 mmol), and 18-crown-6 (10.5 mg, 0.04 mmol) were added to the solution. The solution was refluxed for 12 h at 85 °C. Toluene was removed under reduced pressure, and the residue was purified by silica gel column chromatography (eluting with petroleum ether/ethyl acetate =1/1 until no compound **11** in TLC, change elution to dichloromethane/methanol=10/1), to get **TTB-S1** (121 mg, 56%). **^1^H NMR (400 MHz, DMSO-*d*_6_)** δ 9.18 (d, *J* = 5.0 Hz, 1H), 8.79 (s, 1H), 8.64 (d, *J* = 5.0 Hz, 1H), 8.28 (d, *J* = 5.3 Hz, 1H), 8.19 – 8.08 (m, 2H), 7.98 (d, *J* = 1.8 Hz, 2H), 7.67 (dd, *J* = 5.1, 1.7 Hz, 1H), 7.36 (d, *J* = 8.9 Hz, 2H), 5.78 (s, 2H), 2.82 (d, *J* = 4.8 Hz, 3H). **HRMS** (ESI/TOF) m/z: calculated for C_20_H_17_ClN_5_O_2_S [M+H]^+^ 426.0791 found 426.0779.

**N-(4-chlorophenyl)-7-(pyridin-4-ylmethoxy)thieno[2,3-d]pyridazin-4-amine (TTB-S2)**

Compound **11** (148 mg, 0.5 mmol) and 4-pyridinecarbinol (442 mg, 2.5 mmol) were dissolved in toluene (10mL). KOH (200 mg, 5 mmol), and 18-crown-6 (10.5 mg, 0.04 mmol) were added to the solution. The solution was refluxed for 12 h at 85°C. Toluene was removed under reduced pressure, and the residue was purified by silica gel column chromatography (eluting with dichloromethane/methanol=8/1) to get **TTB-S2** (115 mg, 62%). **^1^H NMR (400 MHz, DMSO-*d*_6_)** δ 9.64 (s, 1H), 8.88 (s, 2H), 8.35 (s, 1H), 8.15 (d, *J* = 5.3 Hz, 1H), 7.98 (d, *J* = 5.4 Hz, 2H), 7.86 (d, *J* = 4.5 Hz, 2H), 7.42 (d, *J* = 6.7 Hz, 2H), 5.87 (s, 2H). **HRMS** (ESI/TOF) m/z: calculated for C_18_H_14_ClN_4_OS [M+H]^+^ 369.0577 found 369.0539.

**4-(((4-((4-Fluorophenyl)amino)thieno[2,3-d]pyridazin-7-yl)oxy)methyl)-N-methylpicolinamide (TTB-S3)**

Compound **12** was synthesized in a similar way to compound **9**, compound **12**, (140 mg, 0.5 mmol) and compound **10** (415 mg, 2.5 mmol) were dissolved in toluene (10 mL). KOH (200 mg, 5 mmol), and 18-crown-6 (10.5 mg, 0.04 mmol) were added to the solution. The solution was refluxed for 12 h at 85°C. Remove toluene under reduced pressure and purified by silica gel column chromatography (eluting with petroleum ether/ethyl acetate =1/1 until no compound **11** in TLC, change elution to dichloromethane/methanol=10/1), to get **TTB-S3** (125 mg, 61%). **^1^H NMR (400 MHz, DMSO-*d*_6_)** δ 9.21 (s, 1H), 8.80 (d, *J* = 5.1 Hz, 1H), 8.64 (d, *J* = 5.0 Hz, 1H), 8.25 (d, *J* = 2.0 Hz, 2H), 8.12 – 8.07 (m, 1H), 7.99 – 7.92 (m, 2H), 7.66 (dd, *J* = 5.0, 1.7 Hz, 1H), 7.14 (t, *J* = 8.9 Hz, 2H), 5.76 (s, 2H), 2.82 (d, *J* = 4.8 Hz, 3H). **^19^F NMR (376 MHz, DMSO-*d*_6_)** δ -121.96. **HRMS** (ESI/TOF) m/z: calculated for C_20_H_17_FN_5_O_2_S [M+H]^+^ 410.1087 found 410.1061.

**Copies of NMR spectra of new compounds**

^
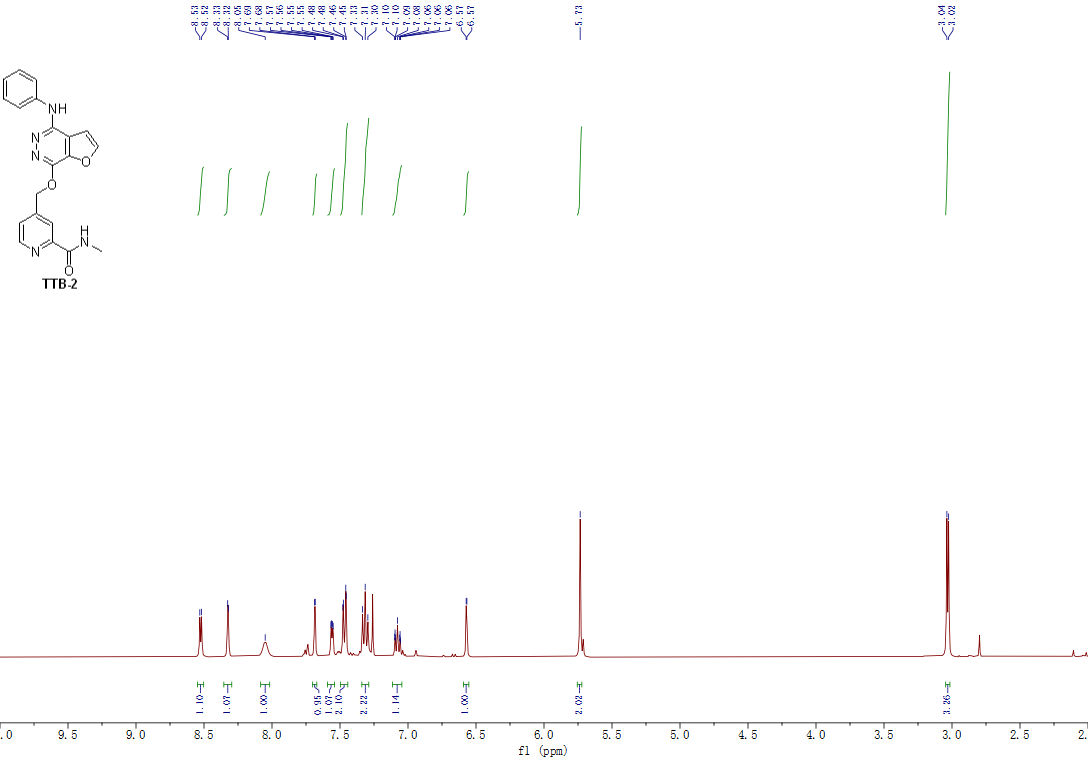
^

^1^H NMR of **TTB-2** (400 MHz, CDCl_3_)


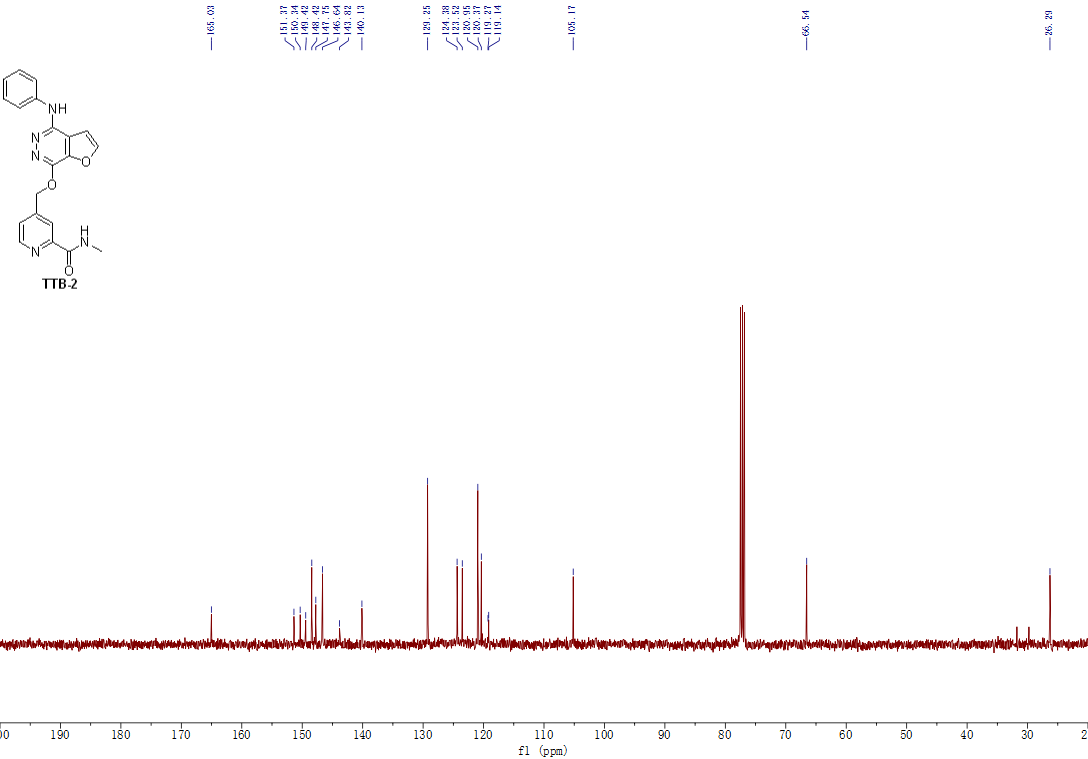


^13^C NMR of **TTB-2** (100 MHz, CDCl_3_)


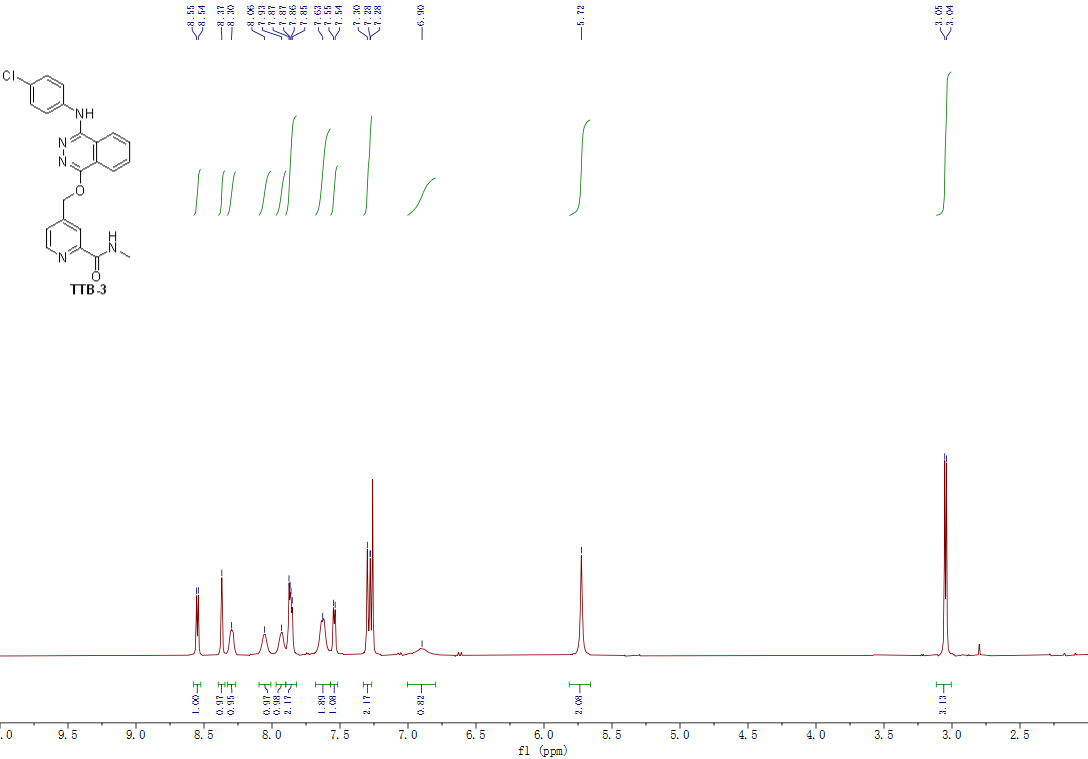


^1^H NMR of **TTB-3** (400 MHz, CDCl_3_)


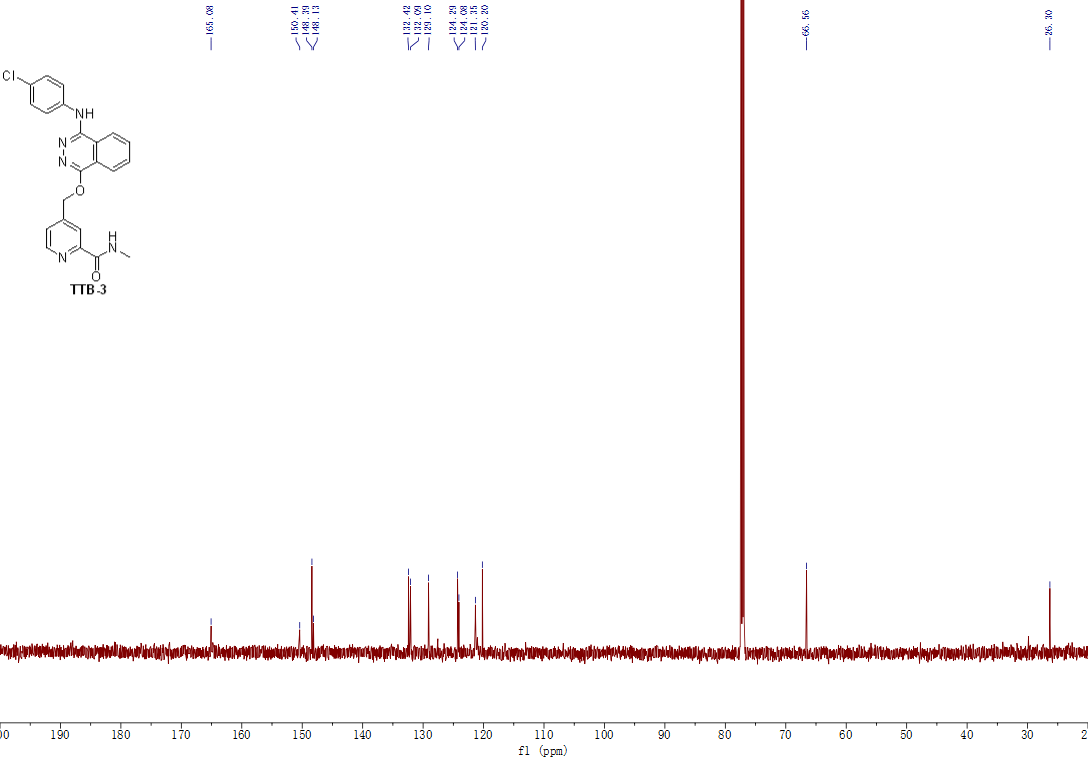


^13^C NMR of **TTB-3** (150 MHz, CDCl_3_)


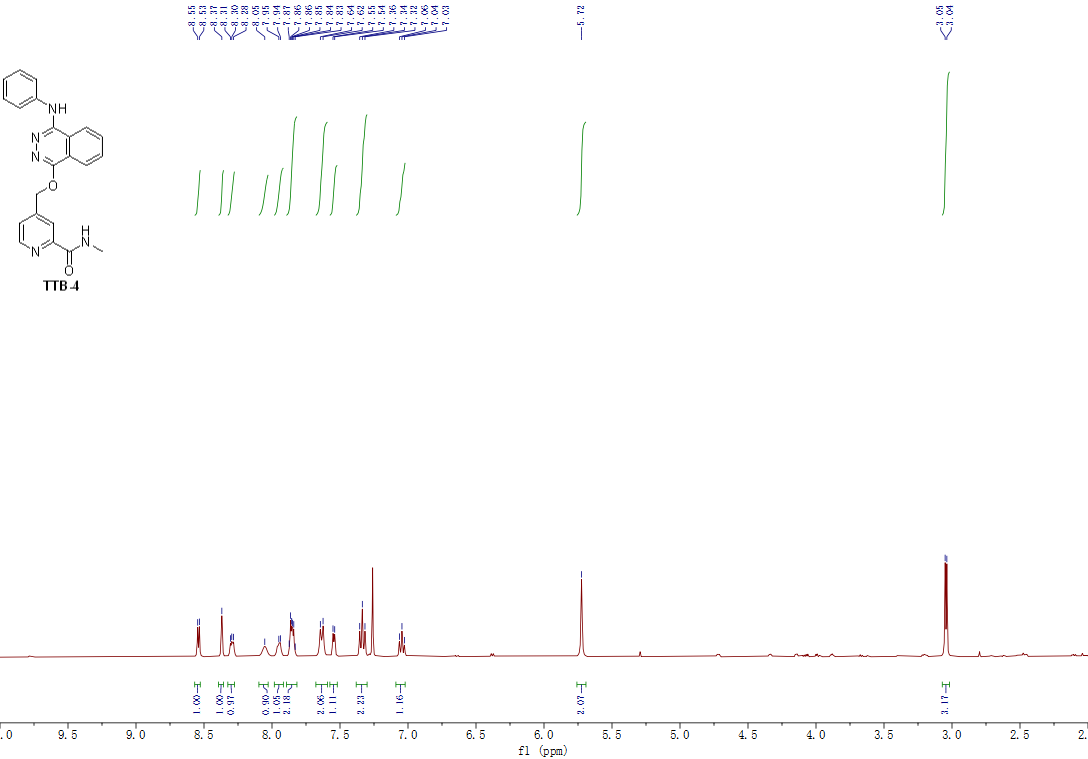


^1^H NMR of **TTB-4** (400 MHz, CDCl_3_)


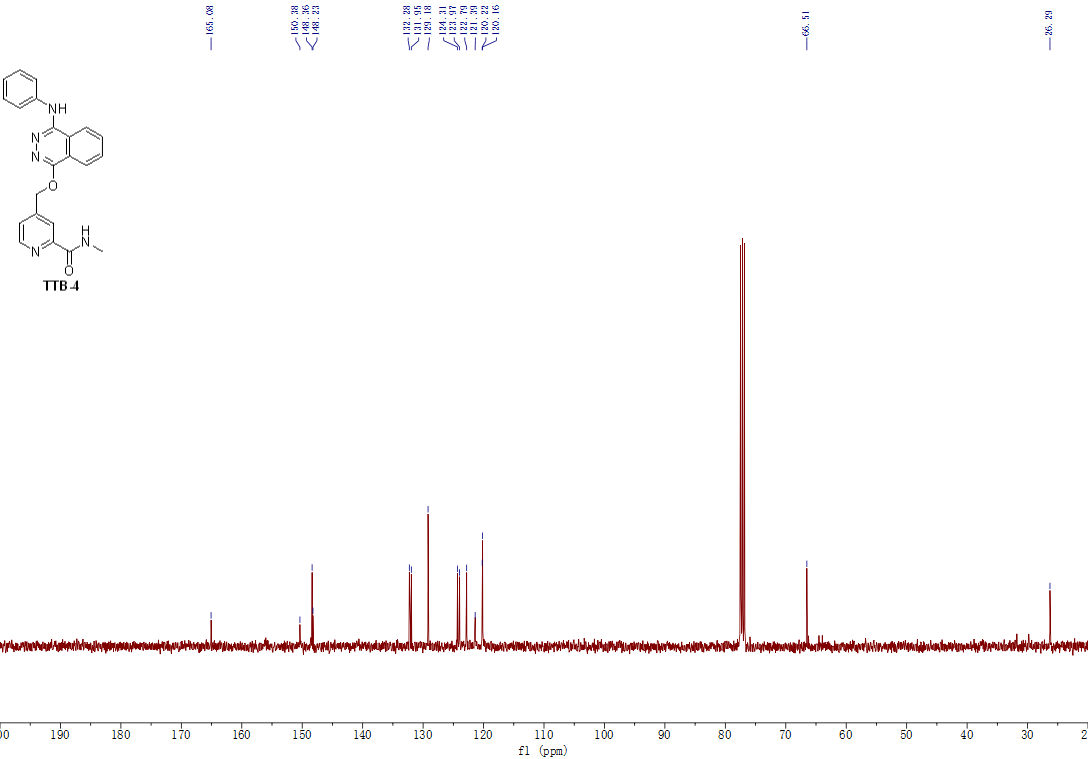


^13^C NMR of **TTB-4** (100 MHz, CDCl_3_)


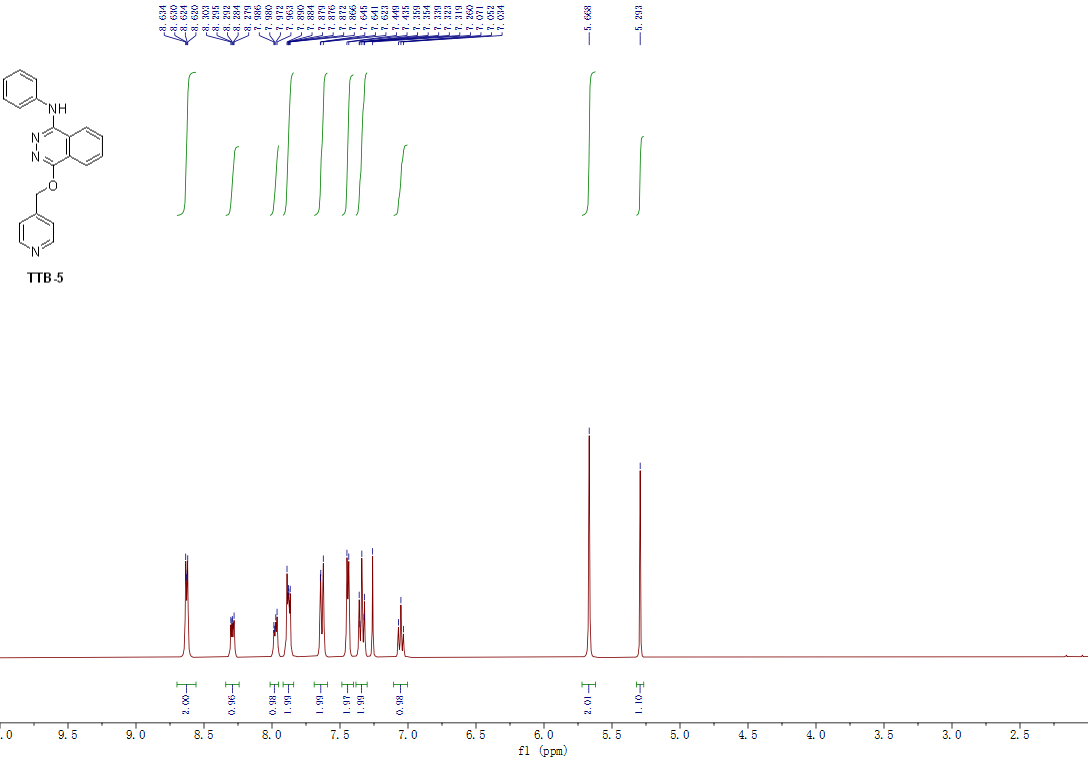


^1^H NMR of **TTB-5** (400 MHz, CDCl_3_)


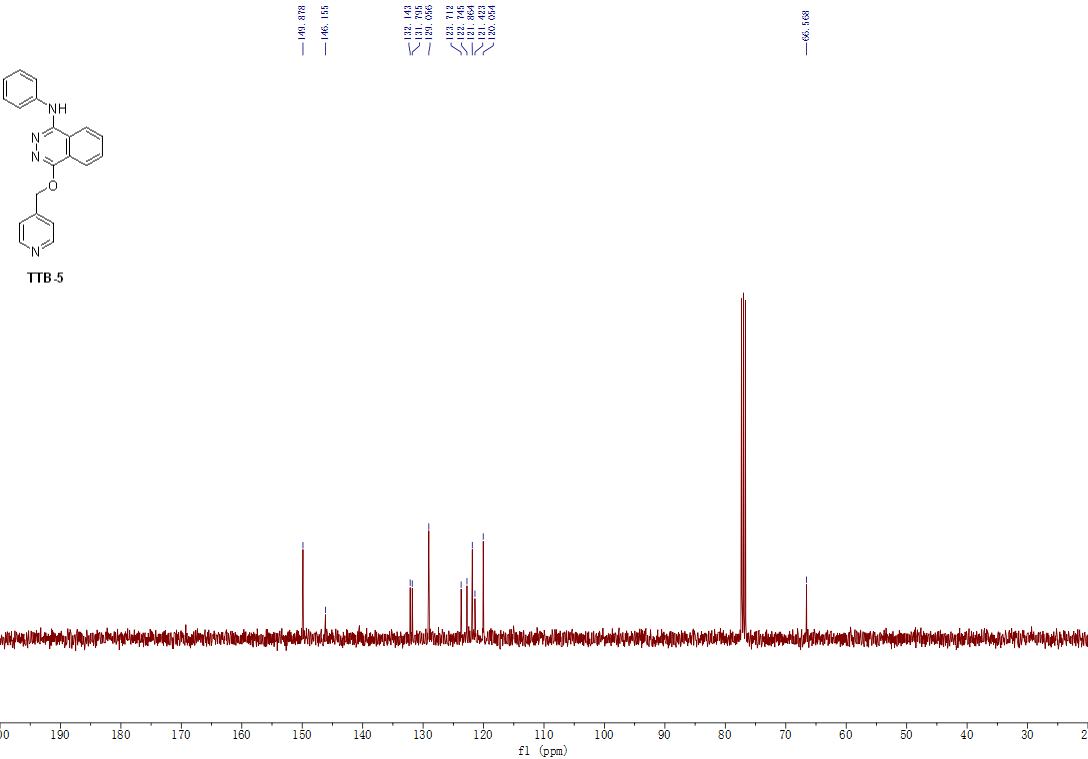


^13^C NMR of **TTB-5** (100 MHz, CDCl_3_)


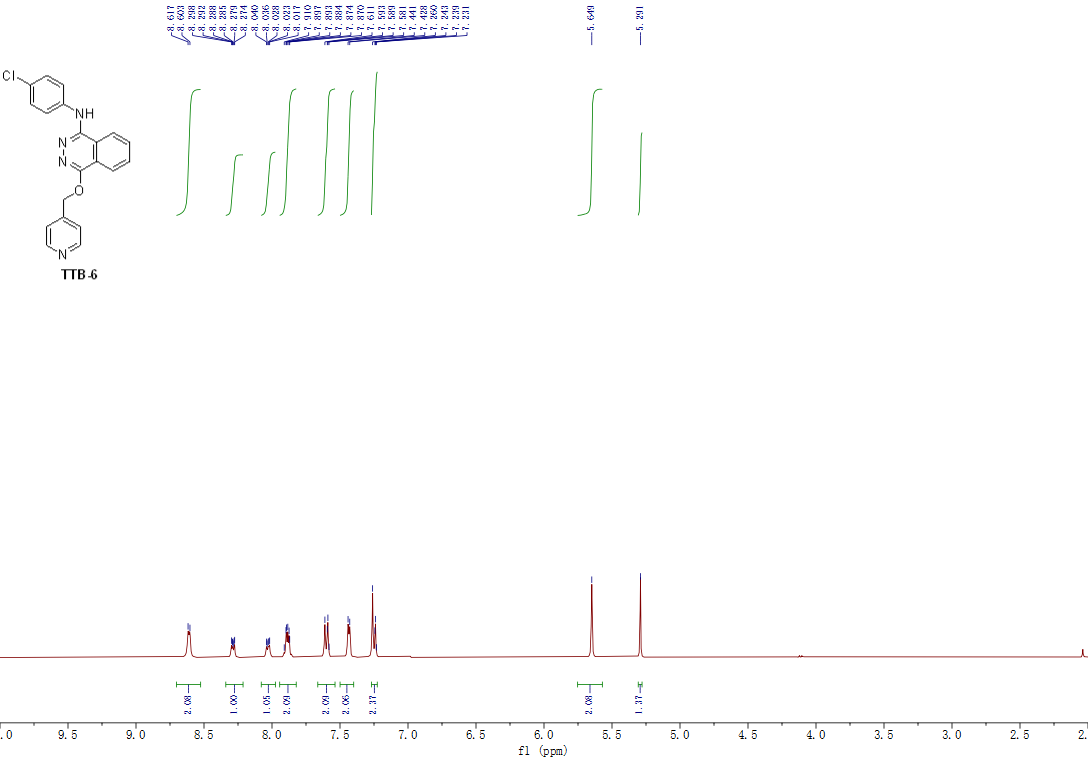


^1^H NMR of **TTB-6** (400 MHz, CDCl_3_)


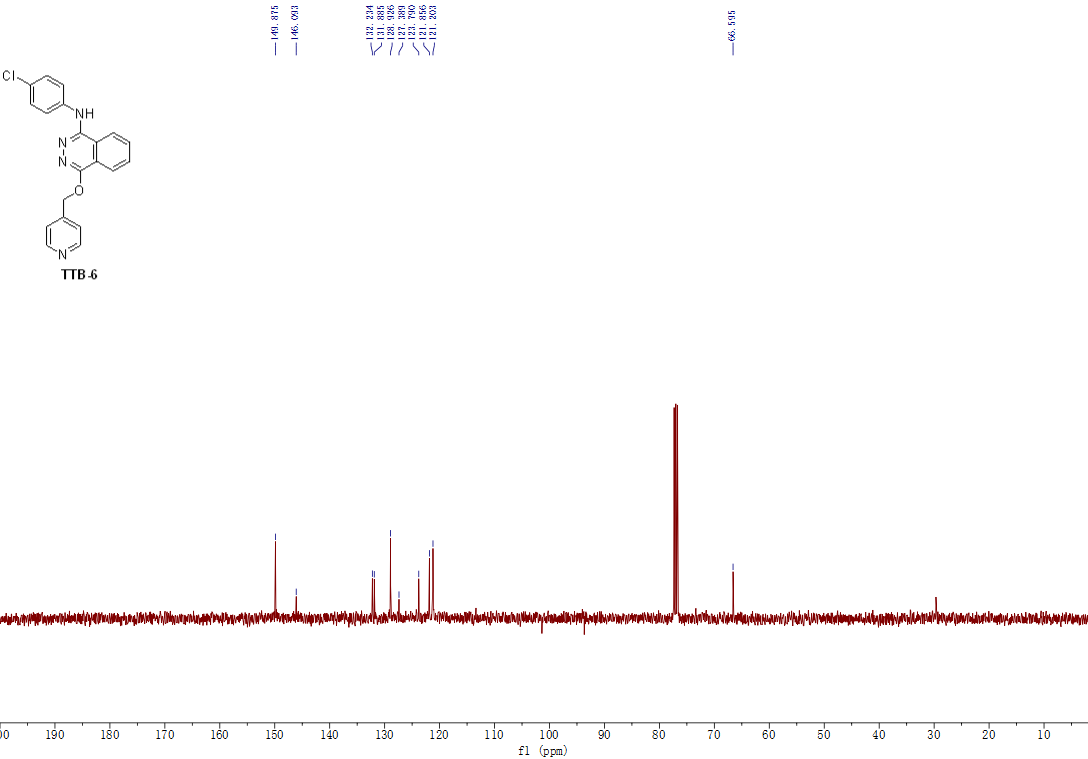


^13^C NMR of **TTB-6** (100 MHz, CDCl_3_)


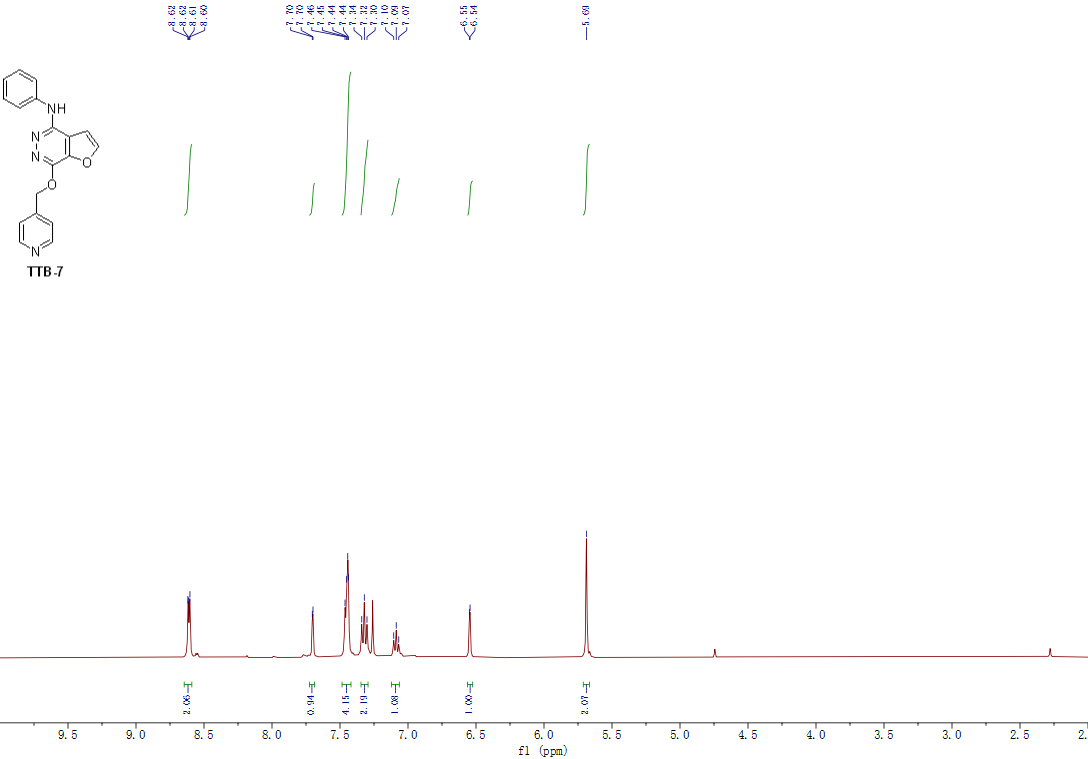


^1^H NMR of **TTB-7** (400 MHz, CDCl_3_)


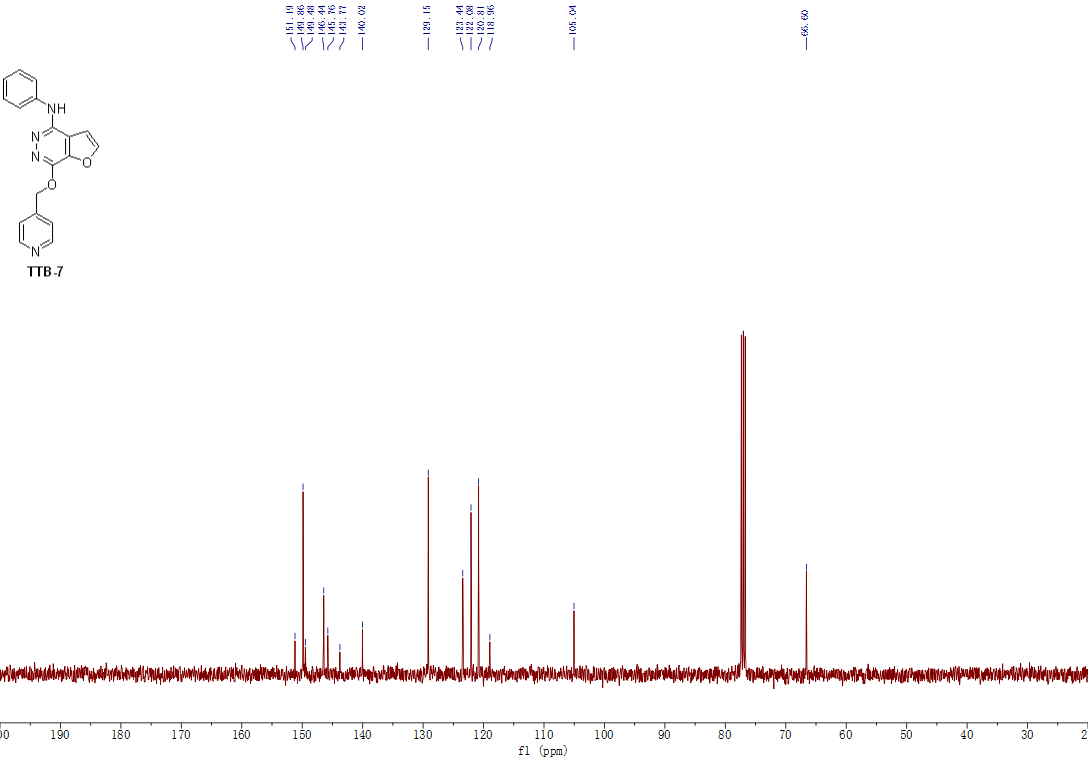


^13^C NMR of **TTB-7** (100 MHz, CDCl_3_)


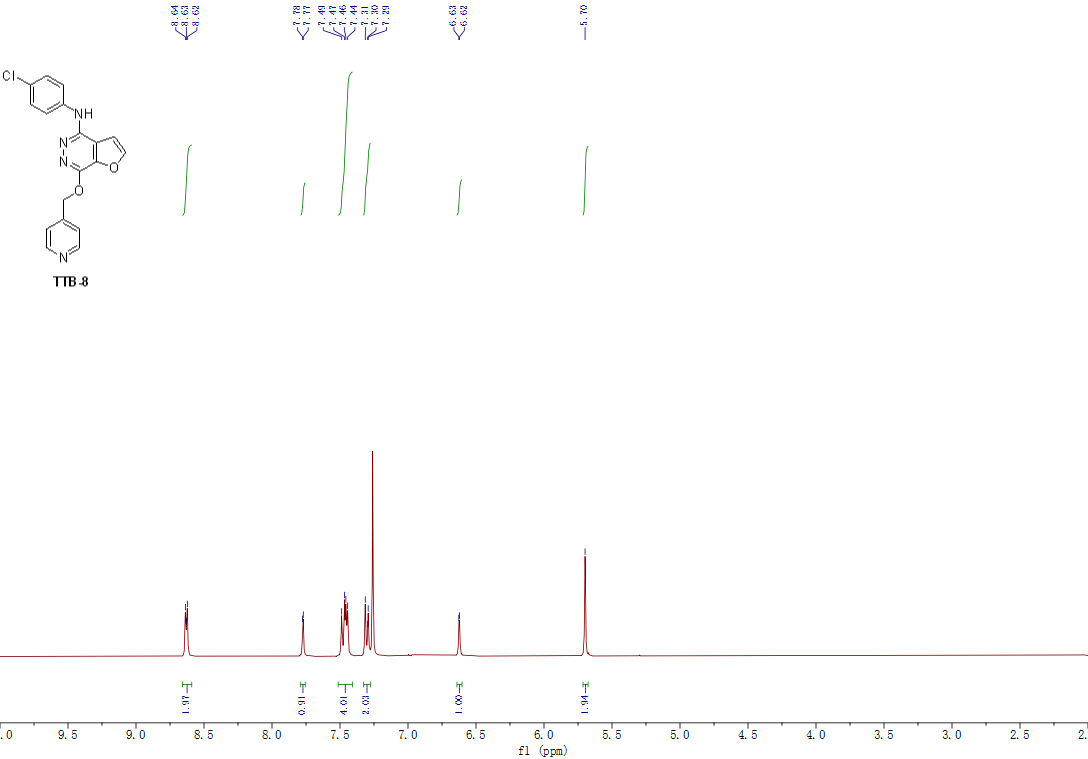


^1^H NMR of **TTB-8** (400 MHz, CDCl_3_)


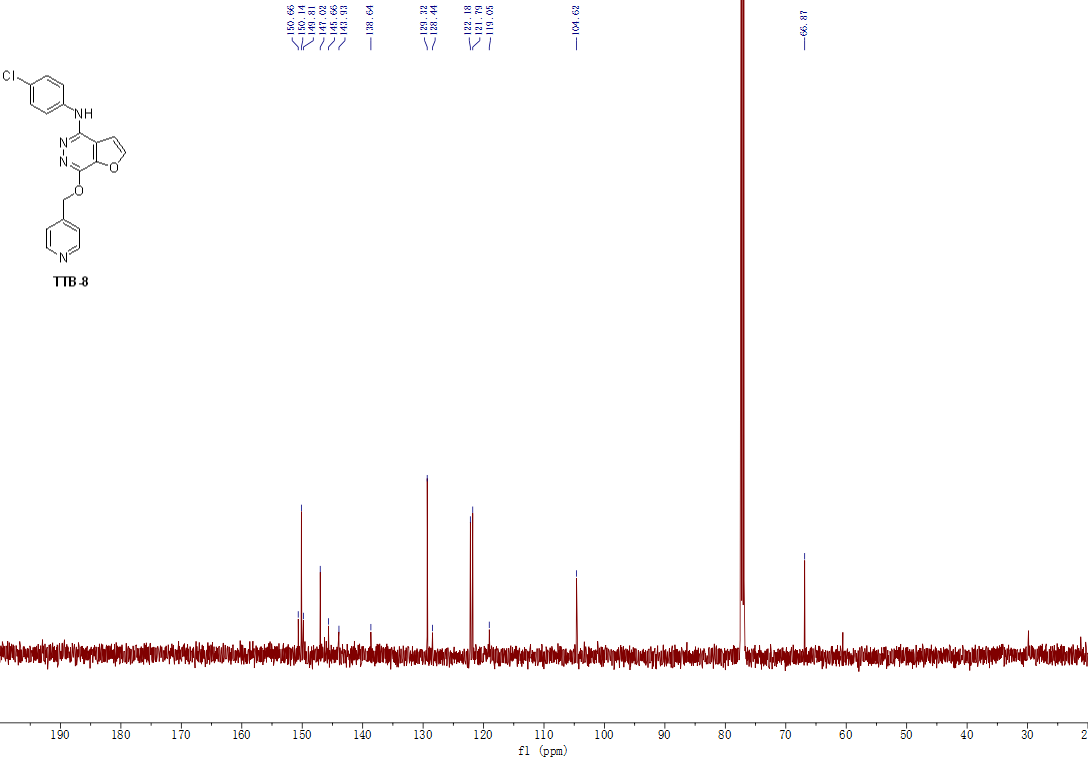


^13^C NMR of **TTB-8** (150 MHz, CDCl_3_)


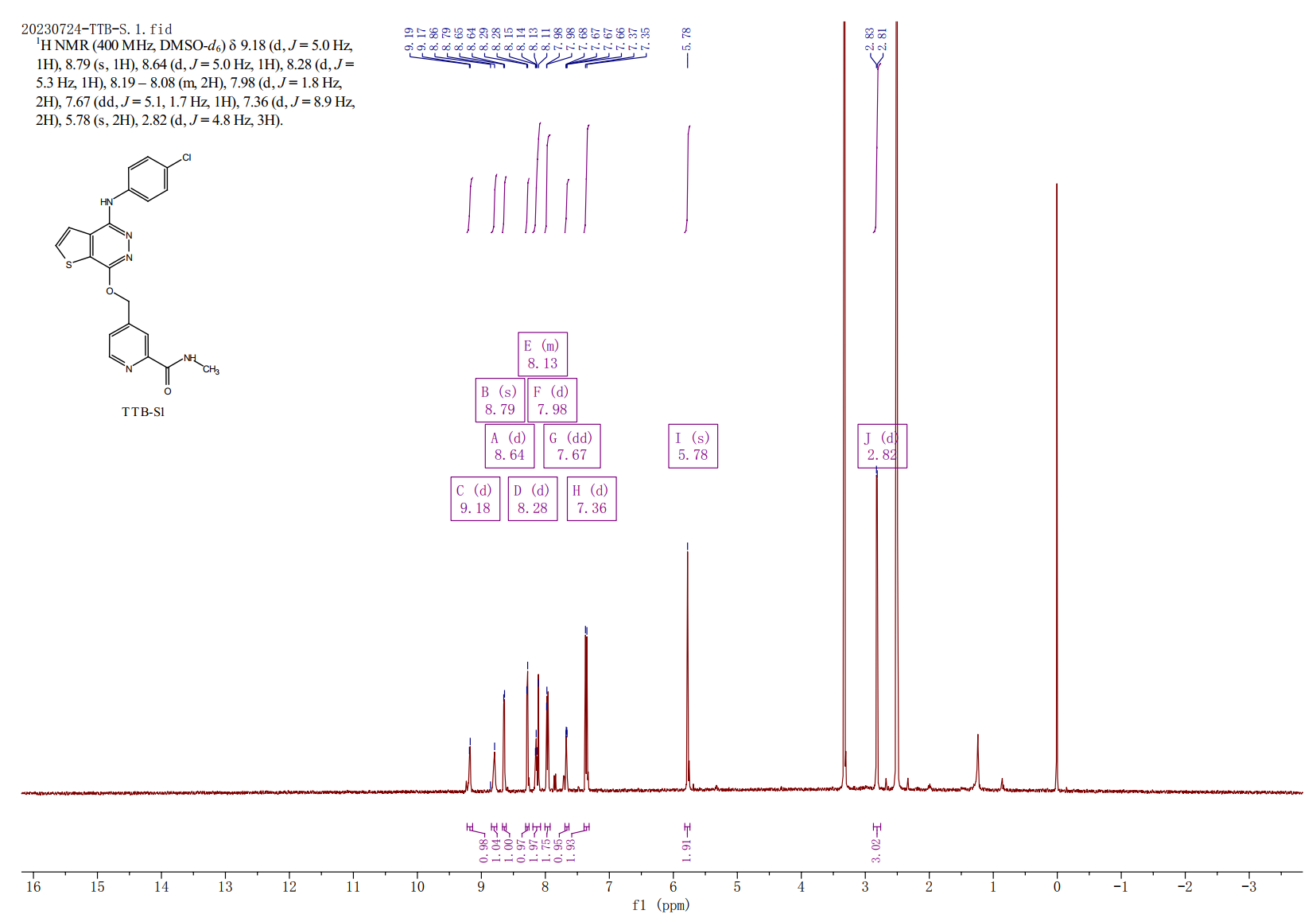
^1^H NMR of **TTB-S1** (400 MHz, DMSO-*d*_6_)


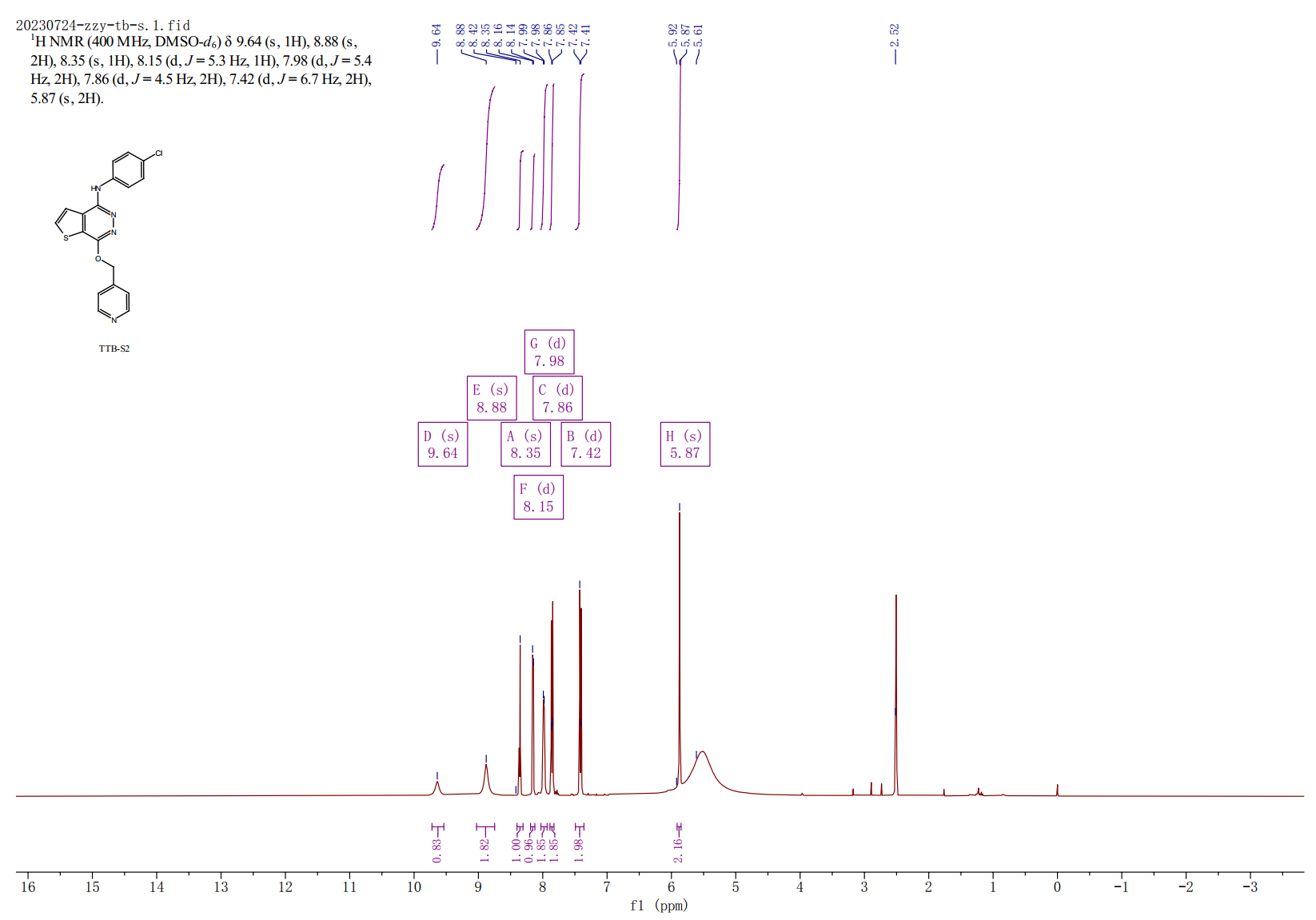


^1^H NMR of **TTB-S2** (400 MHz, DMSO-*d*_6_)


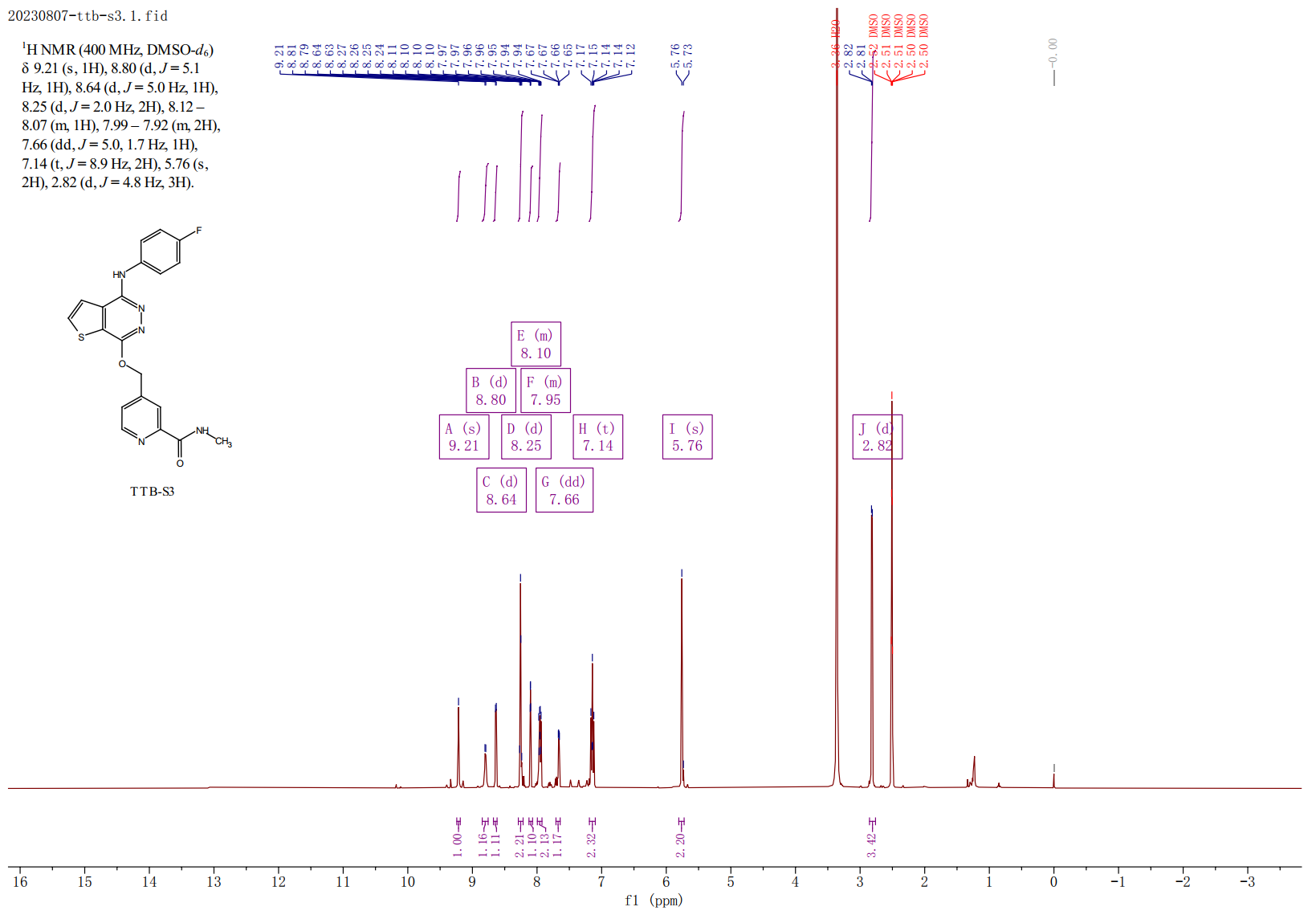


^1^H NMR of **TTB-S3** (400 MHz, DMSO-*d*_6_)


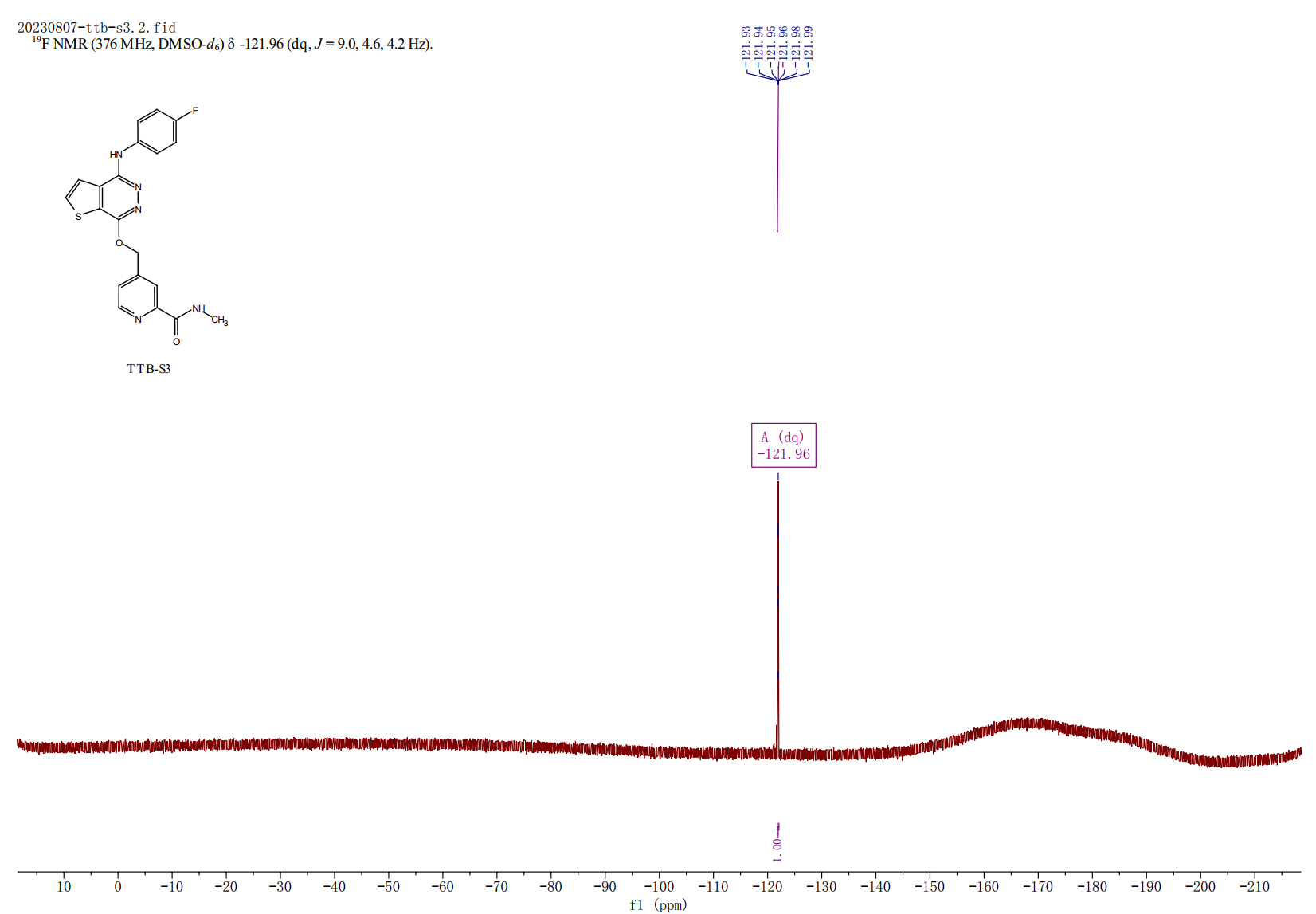


^19^F NMR of **TTB-S3** (376 MHz, DMSO-*d*_6_)

**SI References**

1. Wu, Z., Yan, M., Hu, S. H., Yu, Z. C., Zhu, Y., Cheng, Y. D. *et al.* (2014) Design, synthesis and biological evaluation of indole derivatives as novel inhibitors targeting B-Raf kinase Chinese Chem Lett **25**, 351-354 10.1016/j.cclet.2013.11.006

2. Miller-Moslin, K., Peukert, S., Jain, R. K., McEwan, M. A., Karki, R., Llamas, L. *et al.* (2009) 1-Amino-4-benzylphthalazines as Orally Bioavailable Smoothened Antagonists with Antitumor Activity Journal of Medicinal Chemistry **52**, 3954-3968 10.1021/jm900309j

3. Klein, M., Sandner, P., Frey, R., Riedl, B., andChristensen, O. inventors; Bayer Healthcare Ag (Farb-C) Bayer Healthcare Ag (Farb-C) Bayer Intellectual Property Gmbh (Farb-C) assignee. Use of substituted heteroaryl compounds for manufacturing medicament for treating pulmonary hypertension WO2007118602-A1 25 Oct 2007 A61K-031/435 200865 Pages: 130 English EP2010173-A1 07 Jan 2009 A61K-031/435 200906 English TW200808317-A 16 Feb 2008 A61K-031/4725 200919 Chinese CA2648957-A1 25 Oct 2007 A61K-031/435 200946 English JP2009533479-W 17 Sep 2009 A61K-031/4725 200961 Pages: 106 Japanese US2010113452-A1 06 May 2010 A61K-031/5025 201030 English US8304406-B2 06 Nov 2012 A01N-043/00 201274 English

4. Bollenbach, M., Lugnier, C., Kremer, M., Salvat, E., Megat, S., Bihel, F. *et al.* (2019) Design and synthesis of 3-aminophthalazine derivatives and structural analogues as PDE5 inhibitors: anti-allodynic effect against neuropathic pain in a mouse model Eur J Med Chem **177**, 269-290 10.1016/j.ejmech.2019.05.026

5. Das, S., Ehlers, A. W., Patra, S., de Bruin, B., andChattopadhyay, B. (2023) Iron-Catalyzed Intermolecular C-N Cross-Coupling Reactions via Radical Activation Mechanism J Am Chem Soc **145**, 14599-14607 10.1021/jacs.3c05627
